# Multi-loci phylogeny reveals unexpected novelty of the *Thelephora palmata* complex (Thelephoraceae, Thelephorales) from China

**DOI:** 10.3389/ffunb.2025.1599905

**Published:** 2025-08-14

**Authors:** Tai-Jie Yu, Rou Xue, Lin-Jie Su, Jia Li, Xing Xia, Chang Xu, Han-Chi Lei, Wen-Hao Zhang, Jing Ma, Hong-Yan Huang, Xiu-Juan Li, Yu-Xian Gao, Zhi-Xiang Zhang, Li Li, Guo-Li Zhang, Li-Ping Tang

**Affiliations:** ^1^ School of Pharmaceutical Sciences and Yunnan Key Laboratory of Pharmacology for Natural Products, Kunming Medical University, Kunming, China; ^2^ Yunnan College of Modern Biomedical Industry, Kunming Medical University, Kunming, Kunming, China; ^3^ Key Laboratory of Tropical Translational Medicine of Ministry of Education, School of Pharmacy, Hainan Medical University, Haikou, China; ^4^ School of Pharmacy, Lishui University, Lishui, China; ^5^ Wenshan Center for Disease Control and Prevention, Wenshan, China; ^6^ Jingdong Center for Disease Control and Prevention, Jingdong, China

**Keywords:** ectomycorrhiza, edible mushroom, cryptic species, molecular systematics, taxonomy

## Abstract

*Thelephora palmata* is a well-known species morphologically characterized by coralloid and leathery basidiomata with numerous fuscous purple to blackish-brown branches. It was once considered to exhibit a wide ecological range and distribution area. However, comprehensive phylogenetic analysis based on four loci (ITS, nrLSU, *rpb2*, and nrSSU) revealed that *T. palmata sensu lato* represents a species complex consisting of at least 12 cryptic taxa, with a biogeographic distribution pattern bounded by geographic regions: Asia, Eurasia, Europe, and North America. In this study, we proposed eight new taxa based on available specimens. Of these, seven new species and one forma from China were described here based on phylogenetic analyses, morphological examinations, and environmental niche comparisons, viz., *T. apiculata, T. cornu-damae, T. densa, T. esculenta, T. fuscidula, T. sinopalmata, T. truncicola*, and *T. truncicola * f. *pallescens*.

## 1 Introduction

The genus *Thelephora* Ehrh. ex Willd. was initially proposed by Ehrhart in 1785 (Ehrhart, 1789) and later validly published by Willdenow in 1787 and typified by *T. terrestris* Ehrh. ex Fr (Willdenow, 1787). This genus, along with *Amaurodon* J. Schröt., *Odontia* Pers., *Polyozellus* Murrill., *Tomentella* Pers. ex Pat., and *Tomentellopsis* Hjortstam, belongs to Thelephoraceae, Thelephorales (Larsen, 1968; Reid and Larsen, 1976; Stalpers, 1993; Larsen, 1998; Voitk et al., 2017; He et al., 2024).

Recent molecular phylogenetic analyses, nevertheless, cast doubt on the monophyly of the genus *Thelephora* as previously defined. According to molecular phylogenetic analyses, *Thelephora* has an exceptionally close phylogenetic affiliation with *Tomentella*, and both genera have been generally intermingled on the same evolutionary branches and did not form separate monophyletic groups, suggesting that neither *Thelephora* nor *Tomentella* is monophyletic (Yorou and Agerer, 2008; Lee et al., 2010; Larsson et al., 2019; Svantesson et al., 2021; Villarruel-Ordaz et al., 2024). Therefore, Kõljalg et al. (2024) re-evaluated the limits of *Thelephora* and *Tomentella*, and *Tomentella* was combined into a more comprehensive *Thelephora*.

The reconstituted genus *Thelephora* is characterized macroscopically by annual basidiomata, of two types—resupinate and thin or clavarioid, coralloid to spathulate–rosulate, with various colors and a smooth to granulose surface—and microscopically by subhyaline to brownish, tuberculate, or echinulate basidiospores and monomitic hyphal systems (Fries, 1821; Cunningham, 1963; Corner, 1968, Corner, 1976; Stalpers, 1993; Kõljalg, 1996; Ramírez-López et al., 2015; Tian et al., 2024; Zhang et al., 2024).

This genus is regarded as ectomycorrhizal fungi and is associated with a wide range of angiosperms and gymnosperms, predominantly within Betulaceae, Casuarinaceae, Ericaceae, Fagaceae, and Pinaceae, in a wide range of forest ecosystems, from tropical to temperate, but predominantly temperate (Lentz, 1942; Kõljalg et al., 2000; Roberts and Spooner, 2000; Hilszczanska and Sierota, 2006; Ramírez-López et al., 2013, 2015; Vizzini et al., 2016; Chacón et al., 2018; Das et al., 2018; Liu X. F., et al., 2021; Borovička et al., 2022). Furthermore, some of them hold edible and medicinal values in China. For instance, *Thelephora ganbajun* M. Zang, a traditional edible mushroom with anticancer and anti-allergic effects distributed in Yunnan Province, is favored by local mushroom enthusiasts as one of the most expensive wild mushrooms (Zang, 1987; Hu and Liu, 2001; Sha et al., 2008; Li et al., 2020; Xu et al., 2016; Yang et al., 2023).

*Thelephora palmata* (Scop.) Fr. was originally described based on materials from Europe (Carniola) and morphologically characterized by coralloid and leathery basidiomata with numerous fuscous purple to blackish-brown branches (Fries, 1821; Coker, 1921). This name was also broadly applied to the collections from Asia (Dai, 2011) and North America (Coker, 1921). The wide geographical distribution and broad ecological niches of *T. palmata*, along with the available molecular data, indicate that it could be a species complex consisting of a couple of morphologically similar and phylogenetically related species. In previous studies, these sequences, labeled as “*T. palmata*” from Asia, Europe, and North America, generally clustered within different evolutionary branches and did not form separate monophyletic groups, indicating that this name harbored at least a couple of different taxa in Eurasia and North America (Ramírez-López et al., 2015; Liu X. F., et al., 2021; Yang et al., 2023; Tian et al., 2024).

This study addresses the species diversity of the *T. palmata* complex using an integrative taxonomic approach and describes eight new taxa. Our aim was to gain a comprehensive understanding of the taxonomy of this species complex by examining multiple sources of evidence, including phylogenetic analysis, morphological characteristics, geographical distribution, and host preferences.

## 2 Materials and methods

### 2.1 Collection sites and sampling

A total of 25 specimens of *Thelephora*, including 19 samples so-called *T. palmata*, were collected in the provinces of Anhui, Jilin, and Yunnan, China, from 2019 to 2024 during rainy seasons. These specimens were photographed *in situ* and dried with an electric dryer at 45–50°C after recording their macroscopic features (Hu et al., 2022). The voucher specimens were deposited in the Mycological Herbarium of Kunming Medical University (MHKMU).

### 2.2 Morphological studies

Macroscopic morphology was described according to field notes and photographs of basidiomata, and the color codes referred to those of Kornerup and Wanscher (1978). The micromorphological structures were observed under a light microscope (DM2500, Leica Microsystems, Wetzlar, Germany), which were obtained from dried materials, mounted in 5% KOH and stained with 1% (*w*/*v*) Congo Red solution, when necessary. Melzer’s reagent was used to examine the amyloidity of basidiospores. Basidiospore ornamentations were examined with a ZEISS Sigma 300 scanning electron microscope (Carl Zeiss AG, Oberkochen, Germany). In this paper, [*n*/*m*/*p*] denotes “*n*” basidiospores measured from “*m*” basidiomata of “*p*” collections; (*a*)*b*–*c*(*d*) refers to the length and width of basidiospores, with “*b*–*c*” indicating the distribution interval of 90% of the measured values and “*a*” and “*d*” denoting the minimum and maximum measured values, respectively. “*Q*” is the length/width ratio of a basidiospore in side view, and “Qm” indicates the mean ± standard deviations of the *Q* values of all basidiospores.

### 2.3 DNA extraction, PCR amplification, and sequencing

Extraction of the total genomic DNA from the dried tissue of basidiomata (20 mg) was conducted using the modified CTAB method. Four DNA gene fragments—the nuclear ribosomal DNA internal transcribed spacer (ITS) regions, the large subunit nuclear ribosomal RNA (nrLSU), the RNA polymerase II second largest subunit gene (*rpb2*), and the small subunit of nuclear ribosomal RNA gene (nrSSU)—were amplified by polymerase chain reaction (PCR) using the primer pairs ITS4/ITS5, LR0R/LR5, rpb2-6F/rpb2-7R, and NS1/NS4, respectively (Vilgalys and Hester, 1990; White et al., 1990; Liu S., et al., 2021).

The PCR amplification reaction system included 2.5 μl of 10× PCR buffer (containing MgCl_2_), 0.2 μl Taq DNA polymerase (2.5 U/μl), 0.5 μl of deoxyribonucleotide triphosphate (dNTP, 200 M), 1 μl of each primer (5 μM), and 1 μl template DNA, which was then fixed to 25 μl in sterilized double-steamed water. The reaction system was as follows: pre-denaturation at 94°C for 5 min; 35 cycles of denaturation at 94°C for 40 s, annealing at 52/54/58°C for 40 s, and extension at 72°C for 1 min; and extension at 72°C for 10 min. The PCR conditions followed the description of Tang et al. (2014), Tang et al, 2017). The PCR products were examined by electrophoresis on 1% agarose gels. The amplified PCR products were sequenced using an ABI 3730 DNA Analyzer (Sangon, Shanghai, China) with the same primers. The sequences newly generated in the present study were deposited in GenBank (**Table 1**).

**Table 1:** Information of the DNA sequences used in this study.

Taxa	Voucher	Locality	GenBank accession no.				Reference
			ITS	nrLSU	*rpb2*	nrSSU	
*Odontia fibrosa*	TU115028	–, China	SAMEA4659510	SAMEA4659510	OK632664	SAMEA4659510	Svantesson et al. (2021)
*O. ferruginea*	TUF124098	Estonia	SAMEA4659527	SAMEA4659527	MT724776	SAMEA4659527	GenBank
***Thelephora apiculata***	MHKMU TJ Yu 072^a^	**Anhui, China**	**PQ504921**	**PQ538458**	**PQ560959**	**PQ538484**	**This study**
***T. apiculata***	**MHKMU TJ Yu 072-1**	**Anhui, China**	**PQ504922**	**PQ538459**	**PQ560960**	**–**	**This study**
***T. apiculata***	**MHKMU TJ Yu 242**	**Yunnan, China**	**PQ504919**	**PQ538456**	**PQ560957**	**PQ538482**	**This study**
***T. apiculata***	**MHKMU TJ Yu 245**	**Yunnan, China**	**PQ504920**	**PQ538457**	**PQ560958**	**PQ538483**	**This study**
*T. aquila*	Wei8831	Zhejiang, China	OP793743	OP793698	**–**	**–**	Yang et al. (2023)
*T. aquila*	Wei8833	Zhejiang, China	OP793744	OP793699	**–**	**–**	Yang et al. (2023)
*T. aurantiotincta*	520625MF420	Guizhou, China	MZ057686	**–**	**–**	**–**	GenBank
*T. aurantiotincta*	346-518	Japan	AB509809	**–**	**–**	**–**	GenBank
***T. aurantiotincta***	**MHKMU TJ Yu 041**	**Anhui, China**	**PQ504940**	**PQ538477**	**–**	**–**	**This study**
*T. austrosinensis*	GDGM 48867	Guangdong, China	MF593265	**–**	**–**	**–**	Li et al. (2020)
***T. austrosinensis***	**MHKMU CH Shi 002**	**Anhui, China**	**PQ504935**	**PQ538472**	**PQ560963**	**PQ538497**	**This study**
***T. austrosinensis***	**MHKMU TJ Yu 066**	**Anhui, China**	**PQ504936**	**PQ538473**	**PQ560974**	**–**	**This study**
*T. caryophyllea*	X487	Czech Republic	OL470122	**–**	**–**	**–**	Tian et al. (2024)
*T. caryophyllea*	TL-6566	Denmark	AJ889980	**–**	**–**	**–**	GenBank
***T. cornu-damae***	MHKMU J Ma 005^a^	**Yunnan, China**	**PQ504931**	**PQ538468**	**PQ560969**	**PQ538493**	**This study**
***T. cornu-damae***	**MHKMU J Ma 005-1**	**Yunnan, China**	**PQ504932**	**PQ538469**	**PQ560970**	**PQ538494**	**This study**
*T. dactyliophora*	KUN-HKAS131943	Zhejiang, China	OR940523	**–**	**–**	**–**	Tian et al. (2024)
***T. dactyliophora***	**MHKMU LP Tang 3193**	**Yunnan, China**	**PQ504938**	**PQ538475**	**–**	**–**	**This study**
***T. densa***	MHKMU TJ Yu 293^a^	**Yunnan, China**	**PQ504916**	**PQ538453**	**–**	**PQ538479**	**This study**
***T. densa***	**MHKMU TJ Yu 295**	**Yunnan, China**	**PQ504917**	**PQ538454**	**–**	**PQ538480**	**This study**
***T. esculenta***	MHKMU TJ Yu 243^a^	**Yunnan, China**	**PQ504923**	**PQ538460**	**PQ560961**	**PQ538485**	**This study**
***T. esculenta***	**MHKMU TJ Yu 244**	**Yunnan, China**	**PQ504924**	**PQ538461**	**PQ560962**	**PQ538486**	**This study**
*T. fuscidula* (labeled as *T. palmata*)	TAA149550	Sweden	AF272919	**–**	**–**	**–**	GenBank
***T. fuscidula***	MHKMU HY Huang 359^a^	**Jilin, China**	**PQ504933**	**PQ538470**	**PQ560971**	**PQ538495**	**This study**
***T. fuscidula***	**MHKMU M Mu 357**	**Jilin, China**	**PQ504934**	**PQ538471**	**PQ560972**	**PQ538496**	**This study**
*T. ganbajun*	KUN-HKAS14735	Yunnan, China	KY245240	**–**	**–**	**–**	GenBank
***T. ganbajun***	**MHKMU WH Zhang 770**	**Yunnan, China**	**PQ504939**	**PQ538476**	**–**	**–**	**This study**
*T. glaucoflora*	Dai13623A	Yunnan, China	OP793751	O793696	**–**	**–**	Yang et al. (2023)
***T. glaucoflora***	**MHKMU T Huang 341**	**Yunnan, China**	**PQ504937**	**PQ538474**	**–**	**–**	**This study**
*T. grandinioides*	CLZhao 3406	Yunnan, China	MZ400677	MZ400671	**–**	**–**	Liu X. F., et al. (2021)
*T. grandinioides*	CLZhao 3408	Yunnan, China	MZ400678	MZ400672	**–**	**–**	Liu X. F., et al. (2021)
*T. lacunosa*	KUN-HKAS128968	Yunnan, China	OR512335	**–**	**–**	**–**	Tian et al. (2024)
*T. lacunosa*	KUN-HKAS128966	Yunnan, China	OR512336	**–**	**–**	**–**	Tian et al. (2024)
*T. nebula*	Yuan 11516	Fujian, China	OP793746	OP793694	**–**	**–**	Yang et al. (2023)
*T. nebula*	He 4452	Fujian, China,	OP793748	OP793693	**–**	**–**	Yang et al. (2023)
*T. pacifica*	VO2019_125	Mexico	OR548196	OR602208	**–**	**–**	Ramírez-López et al. (2015)
*T. pacifica*	VO2019_101	Mexico	OR548195	OR602206	**–**	**–**	Ramírez-López et al. (2015)
*T. palmata*	HA27	Latvia	KR019858	**–**	**–**	**–**	GenBank
*T. palmata*	TU115271	Sweden	MH310778	**–**	**–**	**–**	GenBank
“*T. palmata*”-1	JMP0085	USA	EU819443	**–**	**–**	**–**	GenBank
“*T. palmata*”-2	UBC:F33078	Canada	MF908479	**–**	**–**	**–**	GenBank
“*T. palmata*”-2	JLF3733	USA	MK847520	MK847521	**–**	**–**	GenBank
“*T. palmata*”-3	SBK220818_02	Japan	LC800849	**–**	**–**	**–**	GenBank
“*T. palmata*”-3	SBK220707_06	Japan	LC800811	**–**	**–**	**–**	GenBank
“*T. palmata*”-4	CSIRO(M) E7078	Indonesia	AJ537505	**–**	**–**	**–**	GenBank
*T. penicillata*	X619	Prague	OL469899	OL469899	**–**	**–**	Borovička et al. (2022)
*T. penicillata*	X618	Prague	OL469898	OL469898	**–**	**–**	Borovička et al. (2022)
*T. petaloides* (T)	KUN-HKAS97730	Yunnan, China	OR512334	**–**	**–**	**–**	Tian et al. (2024)
*T. petaloides*	KUN-HKAS128970	Yunnan, China	OR512333	**–**	**–**	**–**	Tian et al. (2024)
*T. pinnatifida*	KUN-HKAS96412	Yunnan, China	OR940526	**–**	**–**	**–**	Tian et al. (2024)
*T. pinnatifida*	KUN-HKAS131947	Yunnan, China	OR940525	**–**	**–**	**–**	Tian et al. (2024)
*T. pseudoganbajun*	Yuan 16794	Yunnan, China	OP793768	OP793700	**–**	**–**	Yang et al. (2023)
*T. pseudoganbajun*	Yuan 16835	Yunnan, China	OP793767	OP793702	**–**	**–**	Yang et al. (2023)
*T. pseudoversatilis*	FCME 26232	Mexico	JX075890	**–**	**–**	**–**	Ramírez-López et al. (2015)
*T. pseudoversatilis*	FCME 26152	Mexico	KJ462486	**–**	**–**	**–**	Ramírez-López et al. (2015)
*T. regularis*	S.D. Russell ONT iNaturalist 130285027	USA	OP749556	**–**	**–**	**–**	GenBank
*T. regularis*	bio-material iNAT:177831685	USA	PP400714	**–**	**–**	**–**	GenBank
*T. sikkimensis*	KD 16-003	India	MF684017	**–**	**–**	**–**	Das et al. (2018)
*T. sikkimensis*	KD 16-042	India	MF684018	**–**	**–**	**–**	Das et al. (2018)
***T. sinopalmata***	MHKMU R Xue 190^a^	**Anhui, China**	**PQ504918**	**PQ538455**	**PQ560956**	**PQ538481**	**This study**
*T. terrestris*	Hilszczanska D. 1-IBL	Poland	FJ532478	**–**	**–**	**–**	GenBank
*T. terrestris*	MT1_2203-3	Finland	EU427323	**–**	**–**	**–**	GenBank
***T. truncicola***	**MHKMU LP Tang 2565**	**Yunnan, China**	**PQ504925**	**PQ538462**	**PQ560963**	**PQ538487**	**This study**
***T. truncicola***	**MHKMU T Huang 224-1**	**Yunnan, China**	**PQ504927**	**PQ538464**	**PQ560965**	**PQ538489**	**This study**
***T. truncicola***	MHKMU YJ Pu 097^a^	**Yunnan, China**	**PQ504926**	**PQ538463**	**PQ560964**	**PQ538488**	**This study**
***T. truncicola* f. *pallescens***	**MHKMU Q Deng 31**	**Anhui, China**	**PQ504928**	**PQ538465**	**PQ560966**	**PQ538490**	**This study**
***T. truncicola* f. *pallescens***	**MHKMU LJ Su 408**	**Anhui, China**	**PQ504929**	**PQ538466**	**PQ560967**	**PQ538491**	**This study**
***T. truncicola* f. *pallescens***	MHKMU TJ Yu 255^a^	**Anhui, China**	**PQ504930**	**PQ538467**	**PQ560968**	**PQ538492**	**This study**
*T. versatilis*	UNAM FCME26141	Mexico	NR_154492	**–**	**–**	**–**	Ramírez-López et al. (2015)
*T. versatilis*	UNAM FCME26141	Mexico	KJ462504	**–**	**–**	**–**	Ramírez-López et al. (2015)
*T. vialis*	TENN-F-072094	USA	MN121022	MN121022	**–**	**–**	GenBank
*T. vialis*	TENN-F-072281H2	USA	MN121029	MN121029	**–**	**–**	GenBank
*T. wuliangshanensis*	CLZhao 4107	Yunnan, China	MZ400675	MZ400673	**–**	**–**	Liu X. F., et al. (2021)
*T. wuliangshanensis*	CLZhao 21020	Yunnan, China	MZ400676	MZ400674	**–**	**–**	Liu X. F., et al. (2021)

Newly obtained sequences are shown in bold.

a Holotype; GenBank (two-letter combination), ENA (four-letter combination)

### 2.4 Phylogenetic analyses

Besides the newly generated sequences for this study, additional related sequences downloaded from the GenBank (two-letter combination) and the European Nucleotide Archive (ENA; four-letter combination) were also incorporated into a combined dataset for phylogenetic analyses. Following previous studies, *Odontia ferruginea* Pers. and *O. fibrosis* (Berk. & M.A. Curtis) Kõljalg were chosen as the outgroup taxa (Vizzini et al., 2016; Mu et al., 2021; Tian et al., 2024). The matrices of ITS, nrLSU, *rpb2*, and nrSSU were separately aligned using MUSCLE v3.6 and manually optimized on BioEdit v7.0.9, when necessary (Hall, 1999; Edler et al., 2021). The concatenated datasets were manually constructed.

The combined dataset (ITS + nrLSU + *rpb2* + nrSSU) was analyzed using the maximum likelihood (ML) and Bayesian inference (BI) methods. ML analyses were implemented with raxmlGUI 2.0, and 1,000 rapid bootstrap replicates were performed. GTRGAMMA was set by default as the selected model (Sugawara et al., 2022). For BI analyses, partitioned Bayesian analyses of four Markov chain Monte Carlo (MCMC) chains were run for 5,000,000 generations, with trees sampled every 100 generations until the average standard deviation of split frequencies was <0.01 (Ronquist et al., 2012). The most appropriate substitution models were selected with MrModeltest v2.3 under the Akaike information criterion (AIC) (Nylander, 2004). For the combined dataset, the best-fit likelihood models of ITS, nrLSU, *rpb2*, and nrSSU were HKY+I+G, GTR+I+G, GTR+G, and GTR+I, respectively. The burn-in was set to discard trees sampled from the first 25% of the generations, and then the Bayesian posterior probabilities (PPs) were calculated for a majority consensus tree of the retained Bayesian trees.

## 3 Results

### 3.1 Molecular phylogeny

The combined dataset (ITS + nrLSU + *rpb2* + nrSSU) included 156 sequences (68 from GenBank and 88 newly generated: 25 ITS, 25 nrLSU, 19 *rpb2*, and 19 nrSSU), which were edited and aligned in this study, with 3,374 characters of the alignment length. The alignment was submitted to TreeBASE (accession S31805).

**Figure 1:**
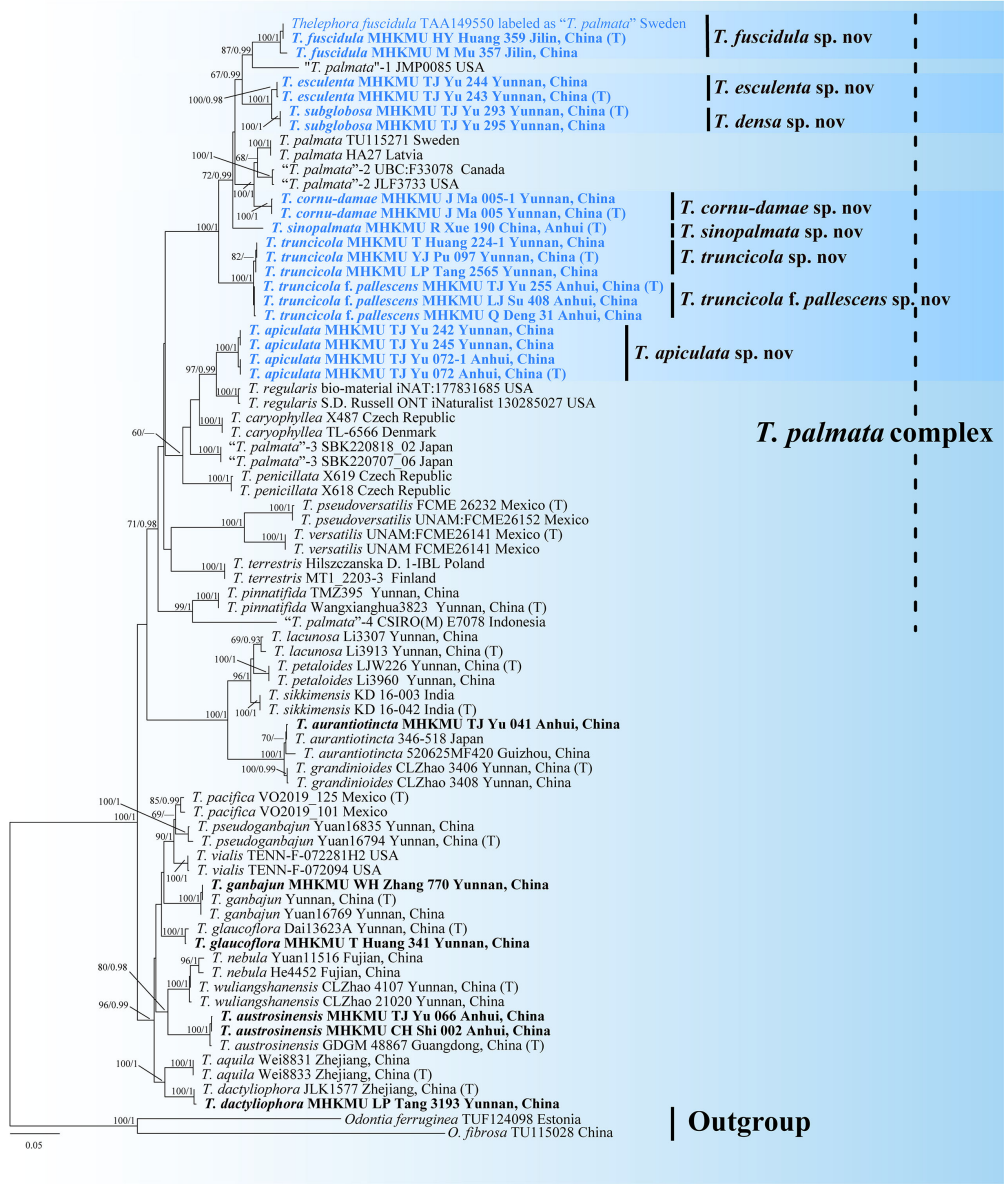
Phylogenetic tree of the genus *Thelephora* based on the ITS + nrLSU + *rpb2* + nrSSU dataset using the maximum likelihood (ML) and Bayesian inference (BI) approaches. ML topology is shown with bootstrap values BS ≥ 50% and Bayesian posterior probabilities PP ≥ 0.9% on the *branches*. Newly generated sequences are in *bold* and new species are in *blue*. *T* represents the holotype.

In the phylogeny, the ML and BI approaches showed minimal differences in the evaluation results; thus, only the ML tree is shown here (**Figure 1**). In a pre-analysis, four DNA gene markers were evaluated: ITS, nrLSU, *rpb2*, and nrSSU. Of these, ITS, nrLSU, and *rpb2* were able to accurately identify different taxa within the *T. palmata* complex. In contrast, nrSSU proved inadequate for distinguishing some close or sibling taxa.

According to the combined molecular tree, the sequences of “*T. palmata*” formed 12 highly supported clades, and these unique lineages had different distributions in Asia, Europe, and North America. Among them, Chinese collections formed eight monophyletic lineages with strong support, corresponding to seven new species and one forma, herein named *Thelephora apiculata*, *Thelephora cornu-damae*, *Thelephora densa*, *Thelephora esculenta*, *Thelephora sinopalmata*, *Thelephora truncicola*, and *Thelephora truncicola* f. *pallescens*, respectively. The “*Thelephora palmata*” from Jinlin, China, and Sweden formed a single branch representing a new species, herein named *Thelephora fuscidula*, which is the only species with a Eurasian distribution in this complex. The remaining sequences of “*T. palmata*” also represented five different taxa: two from North America, one from Europe, one from Indonesia, and one from Japan.

In this study, the sister relationships of some taxa were resolved. The temperate species, *T. fuscidula* from Eurasia, is a sister to “*T. palmata*”-1 from North America (GenBank accession: JMP0085), with moderate support values (BS = 87, PP = 0.99). The subtropical species, *T. apiculata* from Anhui and Yunnan Provinces, China, is a sister to *Thelephora regularis* Schwein. from North America, with strong support values (BS = 97, PP = 0.99). Another subtropical species, *T. cornu-damae* from China, grouped with “*T. palmata*”-2 from Europe and “*T. palmata*”-3 from North America (GenBank accession: JLF3733 and UBC: F33078), with strong support values (BS = 100, PP = 1). Two tropical species from Yunnan Province, China, *T. esculenta* and *T. densa*, formed sister relationship with strong support values (BS = 100, PP = 1). “*T. palmata*”-5 from Indonesia [GenBank accession: CSIRO(M) E7078], representing a tropical species, is a sister to *Thelephora pinnatifida* Yan C. Li & Zhu L. Yang from subtropical regions of China, with strong support values (BS = 99, PP = 1).

### 3.2 Taxonomy

***Thelephora apiculata*** L.P. Tang & T.J. Yu sp. nov.

(**Figures 2A–I**)

**Figure 2:**
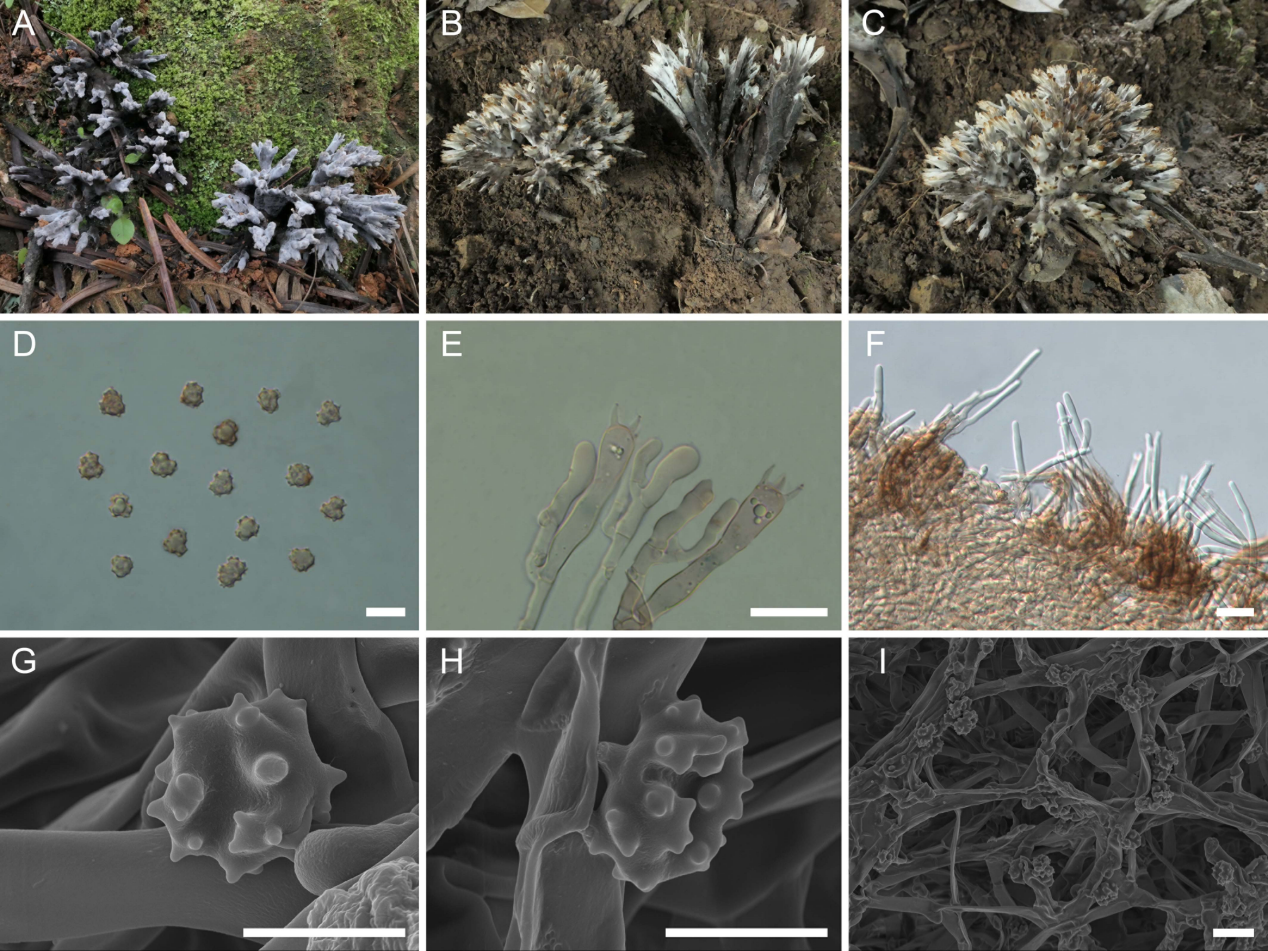
Macroscopic and microscopic features of *Thelephora apiculata*. **(A–C)** Basidiomata: from MHKMU TJ Yu 242 **(A)**, and from MHKMU TJ Yu 072, holotype **(B, C)**. **(D)** Basidiospores. **(E)** Basidia and basidioles. **(F)** Hair-like appendages of pointed tips. **(G–I)** Basidiospores under SEM. *Bars*, 10 µm **(D)**, 20 µm **(E)**, 25 µm **(F)**, 5 µm **(G, H)**, and 10 µm **(I)**.

**MycoBank:** 856466

**Etymology:** *apiculata* (Latin), refers to the conical and acute terminals of branches.

**Diagnosis**: Distinct from other species within the *T. palmata* complex in having grayish to grayish-black tomentose to velvety branches, with grayish-white, conical, and acute terminals, short to insignificant stipe, relatively small basidiospores (6.2–7.1 µm × 5.9–6.7 µm) with short echinulis measuring 0.3–0.5 µm, narrower basidia (46–62.8 µm × 7–10.5 µm), and occurrence under trees of *Castanea mollissima* and *Quercus* spp. in subtropical forests.

**Type:** China—Anhui Province, Shitai County, Dayan Town, Guniujiang National Nature Reserve, 30°04'51" N, 117°28'25" E, elevation 520 m, July 12, 2023, on the ground of broad-leaved forests dominated by *Castanea* spp. and *Quercus* spp., Taijie Yu 072 (MHKMU TJ Yu 072, holotype).

**Description:** Basidiomata solitary, scattered to gregarious, upright, coralloid, and merismatoid, in tufts 5–7 cm high and 3–5 cm broad, coriaceous and moist when fresh, while corky and light in weight when dry; branches arising from a shared center or stipe, branched in three to four ranks, subcylindrical, clavarioid to conical, tomentose to velvety, 0.5–1 cm wide; terminal and margin thin, 0.3–0.5 mm thick, acute and pointed, usually moderately lacerate, forming a small and short sawtooth. Hymenial surface tomentose to velvety, rugulose, azonate, whitish (1A2) to pale grayish white (2B2) initially, dark gray (1E1) to nearly black (2F2) when mature, but pale grayish-white (2B2) at margin, whitish (1A2) after drying. Context 2–5 mm thick, relatively thin at the margin and thick toward the base, gray-brown (4D2). Stipe concolourous with hymenial surface, (0–) 0.2–1.5 cm long, (0–) 0.2–0.6 cm in diameter, irregularly cylindrical to flattened or broadened at the base, surface tomentose, slightly rugose. Odor mild when fresh, slightly sweet and fragrant upon drying. Taste not recorded.

Hymenium predominantly amphigenous. Basidiospores [60/4/2] (6–) 6.2–7.1 (–7.8) × (5.7–) 5.9–6.7 (–7) µm (ornamentations excluded), *Q* = 1.01–1.15 (–1.37), Qm = 1.08 ± 0.07, globose to subglobose in frontal and lateral views, irregular in outline, slightly thick-walled, inamyloid, pale brownish in 5% KOH, and yellow-brown to brownish in Melzer’s reagent; surface nodulose to verrucose, echinulis numerous and prominent, up to 0.3–0.5 µm high, usually isolated, sometimes in groups of two or three, then bifurcate in shape, subconical, but apex somewhat obtuse; hilar appendage oblique. Basidia 46–62.8 (–74) × 7–10.5 µm, narrow clavate, four-spored, clamped at base, occasionally with finely granulose contents; sterigmata 5–8 µm long. Basidioles numerous, similar to basidia in shape, but slightly smaller. Cystidia absent. Hyphal structure: Hyphal system monomitic, generative hyphae with clamp connections. Hymenophoral trama composed of hyphae in a subparallel to an interwoven pattern; hyphae 2–3.5 µm wide, clamped, moderately branched, generally branched near clamp connections, occasionally flexuous and collapsed. Hair-like appendages of pointed margin composed of subparallel and loosely interwoven hyphae; hyphae cylindrical, thin-walled, clamped, occasionally branched, generally branched near clamp connections, 3–4 µm wide, terminal cell rounded at margin. Clamp connections abundant on hyphae of all tissues.

**Habitat:** Solitary, scattered to gregarious on the ground of subtropical mixed coniferous–broad forests dominated by broad-leaved trees, *C. mollissima*, *Quercus* spp., and a few conifers, *Keteleeria evelyniana*.

**Additional specimens examined:** China—Anhui Province, Shitai County, Dayan Town, Guniujiang National Nature Reserve, 30°04'51" N, 117°28'25" E, elevation 520 m, July 12, 2023, on the ground of broad-leaved forests dominated by *Castanea* spp. and *Quercus* spp., Taijie Yu 072-1 (MHKMU TJ Yu 072-1). Yunnan Province, Guangnan County, Dongbao Town, Nongdang, 25°12'15" N, 105°05'31" E, elevation 1,400 m, July 7, 2024, on the ground of mixed coniferous–broad forests dominated by *C. mollissima* and a few *K. evelyniana*, Taijie Yu 242 (MHKMU TJ Yu 242); same location and date, Taijie Yu 245 (MHKMU TJ Yu 245).

**Known distribution:** Currently known from Anhui and Yunnan Provinces, China.

**Notes:** Morphologically, the *T. apiculata* from the subtropical regions of China is recognized by its tomentose to velvety branches with conical and acute terminals, short to insignificant stipes, and small basidiospores (6.2–7.1 μm × 5.9–6.7 µm) with short echinulis, up to 0.3–0.5 µm. In terms of basidiomatal color, *T. apiculata* closely resembles *T. sinopalmata*. Both of them are from the subtropics of China, but the latter is distinguished by its shorter and slender branches, with blunt and round terminals, and larger basidiospores measuring 8.2–10.4 μm × 6.8–8.7 μm. Furthermore, *T. apiculata* resembles *T. cornu-damae*, *Thelephora dactyliophora* Yan C. Li & Zhu L. Yang, *Thelephora pseudoversatilis* Ramírez-López & M. Villegas, and *Thelephora versatilis* Ramírez-López & M. Villegas. However, the *T. cornu-damae* from China is distinguished by its longer basidiomata, with flattened and wide terminals, usually deeply lacerate and serrulate, longer and significant stipe, and relatively larger basidiospores measuring 8.6–10 µm × 7–8.4 µm, with longer echinulis (1.2–1.5 µm). *T. dactyliophora*, distributed in Yunnan and Zhejiang Provinces, is distinguished by its flattened and thinner branches with lacerate margin, brownish-gray to gray hymenial surface, and orange-white to gray-white context (Tian et al., 2024). *T. pseudoversatilis*, originally described from Mexico, is distinguished by its brownish-gray to violet-brown branches, longer basidiospores measuring 7−8 μm × 5.5−6 μm, with longer echinulis, up to 1–2 µm, and tropical distribution. Another Mexican species, *T. versatilis*, is distinguished by its longer basidiomata, up to 9.5 cm, with brownish, flattened, and wider branches, longer echinulis, up to 1–1.5 µm, and tropical distribution (Ramírez-López et al., 2015).

Phylogenetically, *T. apiculata* formed a sister relationship with *T. regularis*. However, *T. regularis*, originally described from North Carolina, USA, is distinguished by its reddish or rusty-buff, infundibuliform or rarely subdimidiate, and smaller basidiomata, buff-colored hymenial surface with a pinkish tinge, longer basidiospores measuring 7.5−8.75 µm × 4.5−6 µm, and temperate distribution in North America (Reid, 1962; Corner, 1968).

***Thelephora cornu-damae*** L.P. Tang & T.J. Yu sp. nov.

(**Figures 3A–I**)

**Figure 3:**
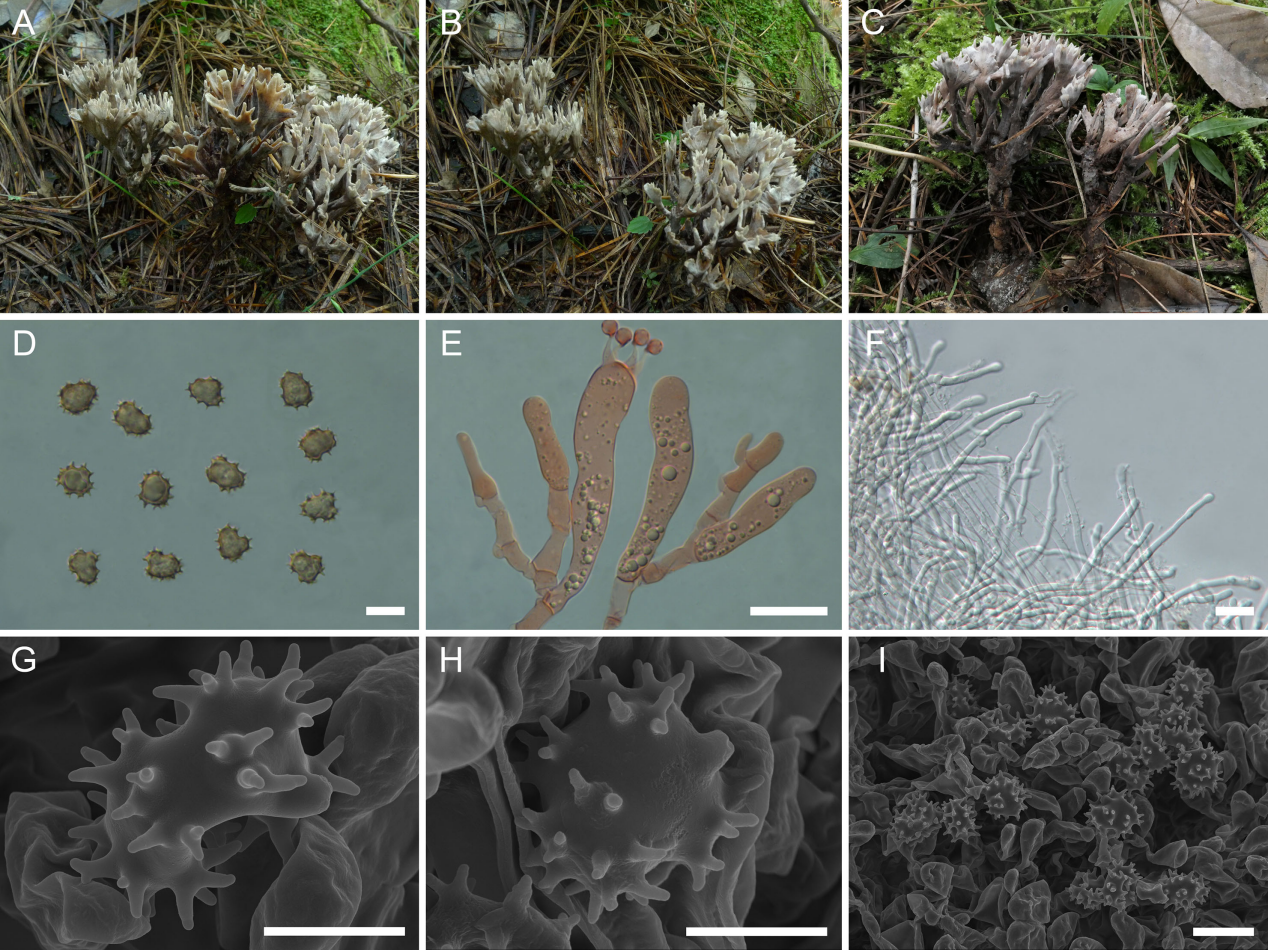
Macroscopic and microscopic features of *Thelephora cornu-damae* (MHKMU J Ma 005, holotype). **(A–C)** Basidiomata. **(D)** Basidiospores. **(E)** Basidia and basidioles. **(F)** Hair-like appendages of pointed tips. **(G–I)** Basidiospores under SEM. *Bars*, 10 µm **(D)**, 20 µm **(E)**, 25 µm **(F)**, 5 µm **(G, H)**, and 10 µm **(I)**.

**MycoBank:** 856467

**Etymology:** *cornu-damae* (Latin), refers to the elkhorn-like branches of this species.

**Diagnosis**: Distinct from other species within the *T. palmata* complex in having pale grayish-white branches, with wide spathulate or flabelliform, deeply lacerate and serrulate terminals, relatively long and significant stipe, covered with granular papillate, relatively large and ellipsoid basidiospores (8.6–10 µm × 7–8.4 µm) with long echinulis, up to 1.2–1.5 µm, and occurrence under *Pinus yunnanensis* in subtropical forests.

**Type:** China—Yunnan Province, Qujing City, Haizhai Forestry Area, 25°15'01" N, 103°59'19" E, elevation 2,100 m, October 1, 2021, on the ground of mixed coniferous–broad forests dominated by *P. yunnanensis* and a few *Rhododendron* spp. and *Quercus* spp., Jing Ma 005 (MHKMU J Ma 005, holotype).

**Description:** Basidiomata solitary, scattered to gregarious, upright, coralloid, and merismatoid, in tufts 6–8 cm high and 4–6.5 cm broad, coriaceous and moist when fresh, while firm, brittle, and light in weight when dry; branches arising from a shared stipe, branched in four to five ranks, flattened, finger-like to narrow flabelliform initially, wide spathulate to flabelliform or lobate when mature, subglabrous, 0.4–0.6 cm wide; terminal thin, 0.4–0.6 mm thick, deeply lacerate and serrulate, forming an elkhorn shape, whitish (1A1). Hymenial surface subglabrous, sometimes papillate, pale grayish-white (2B1) to gray (1C2) initially, darkening to brownish-gray (3D2) to grayish-brown (3A2) with age, nearly smooth to somewhat radially rugulose or wrinkled, azonate. Context 2–3 mm thick, relatively thin at the margin and thick toward the base, brown-gray (2D2). Stipe 3–4 cm in length and 0.5–0.7 cm in diameter, irregularly flattened or slightly broadened at base, surface rugose, with granular papillate, dark gray (1E2) to grayish-brown (3D1). Odor mild when fresh, but strong and agreeable upon drying. Taste not recorded.

Hymenium predominantly amphigenous. Basidiospores [80/4/2] (8.3–) 8.6–10 (–10.4) × (6.7–) 7–8.4 (–9.2) µm (ornamentations excluded), *Q* = (1.01–) 1.07–1.43 (–1.44), Qm = 1.24 ± 0.10, subglobose to ellipsoid in frontal and lateral views, slightly thick-walled, inamyloid, brown in 5% KOH, and yellow-brown to brown in Melzer’s reagent; surface echinulate, echinulis numerous and prominent, up to 1.2–1.5 µm high, usually in groups of two, then bifurcate in shape, subconical, but apex somewhat obtuse; hilar appendage oblique. Basidia 62–74.5 µm × 9.5–14.5 µm, clavate, four-spored, clamped at base, usually with finely granulose contents; sterigmata 6.5–9.7 µm long. Basidioles numerous, similar to basidia in shape, but slightly smaller. Cystidia absent. Hyphal structure: Hyphal system monomitic, generative hyphae with clamp connections. Hymenophoral trama composed of hyphae in a subparallel to an interwoven pattern; hyphae 3.5–5 µm wide, clamped, moderately branched, generally branched near clamp connections, occasionally flexuous and collapsed. Hair-like appendages of pointed margin composed of subparallel and loosely interwoven hyphae; hyphae cylindrical, thin-walled, clamped, occasionally branched, generally branched near clamp connections, 3.5–4.5 µm wide, terminal cell rounded at margin. Clamp connections abundant on hyphae of all tissues.

**Habitat:** Solitary, scattered to gregarious on the ground of subtropical mixed coniferous–broad forests dominated by *P. yunnanensis* and a few *Quercus* spp. and *Rhododendron* spp.

**Additional specimens examined:** China—Yunnan Province, Qujing City, Haizhai Forestry Area, 25°15'01" N, 103°59'19" E, elevation 2,100 m, October 1, 2021, on the ground of mixed coniferous–broad forests dominated by *P. yunnanensis* and a few *Quercus* spp. and *Rhododendron* spp., Jing Ma 005-1 (MHKMU J Ma 005-1).

**Known distribution:** Currently known from Yunnan Province, China.

**Notes:** Morphologically, the *T. cornu-damae* from the subtropical regions of China is recognized by its grayish-white and wide spathulate or flabelliform terminals, relatively long and significant stipe, and ellipsoid basidiospores with long echinulis, up to 1.2–1.5 µm. In terms of basidiomatal color, *T. cornu-damae* is similar to several species from China, such as *T. apiculata*, *T. dactyliophora*, and *T. pinnatifida*. Of these, *T. apiculata* is distinguished by its velvety to tomentose basidiomata, round branches with acute whitish terminals, grayish-black short stipe, smaller basidiospores measuring 6.2–7.1 μm × 5.9–6.7 μm, with shorter echinulis approximately 0.3–0.5 μm, and a strong odor upon drying. *T. dactyliophora* is distinguished by its chalky-white to orange-white and thinner branches, relatively shorter and indistinctive stipe, smaller basidiospores measuring 5–7.5 μm × 4–6.5 μm, with shorter echinulis approximately 0.3–0.5 μm, and a sweet odor upon drying (Tian et al., 2024). *T. pinnatifida* has a geographically overlapping distribution with *T. cornu-damae* in Yunnan Province. However, *T. pinnatifida* is distinguished by its brownish-orange to brown branches with orange-white and needle-like margin, smaller basidiospores measuring 6–9.5 μm × 5–8.5 μm, with shorter echinulis approximately 0.3–0.6 μm, and a yeast powder flavor on drying (Tian et al., 2024). Furthermore, *T. cornu-damae* is reminiscent of *Thelephora penicillata* (Pers.) Fr. However, *T. penicillata* is fairly common in wet acidic conifer plantations throughout Europe and North America and is distinguished by its purplish-brown stipe base and white or cream branches, turning brown from the center with age (Corner, 1968).

Phylogenetically, *T. cornu-damae* has an exceptionally close phylogenetic affiliation with “*T. palmata*”-2 from Europe (Latvia and Sweden) and “*Thelephora palmata*”-3 from North America (Canada and USA). However, *T. palmata* is distinguished by its fuscous purple, chocolate-brown to blackish-brown basidiomata, with flattened, palmatifid, multifid, or dichotomous branches and longer basidiospores measuring 8–12 µm × 6–8 µm (Fries, 1821; Coker, 1921).

***Thelephora densa*** L.P. Tang & T.J. Yu sp. nov.

(**Figures 4A–F**)

**Figure 4:**
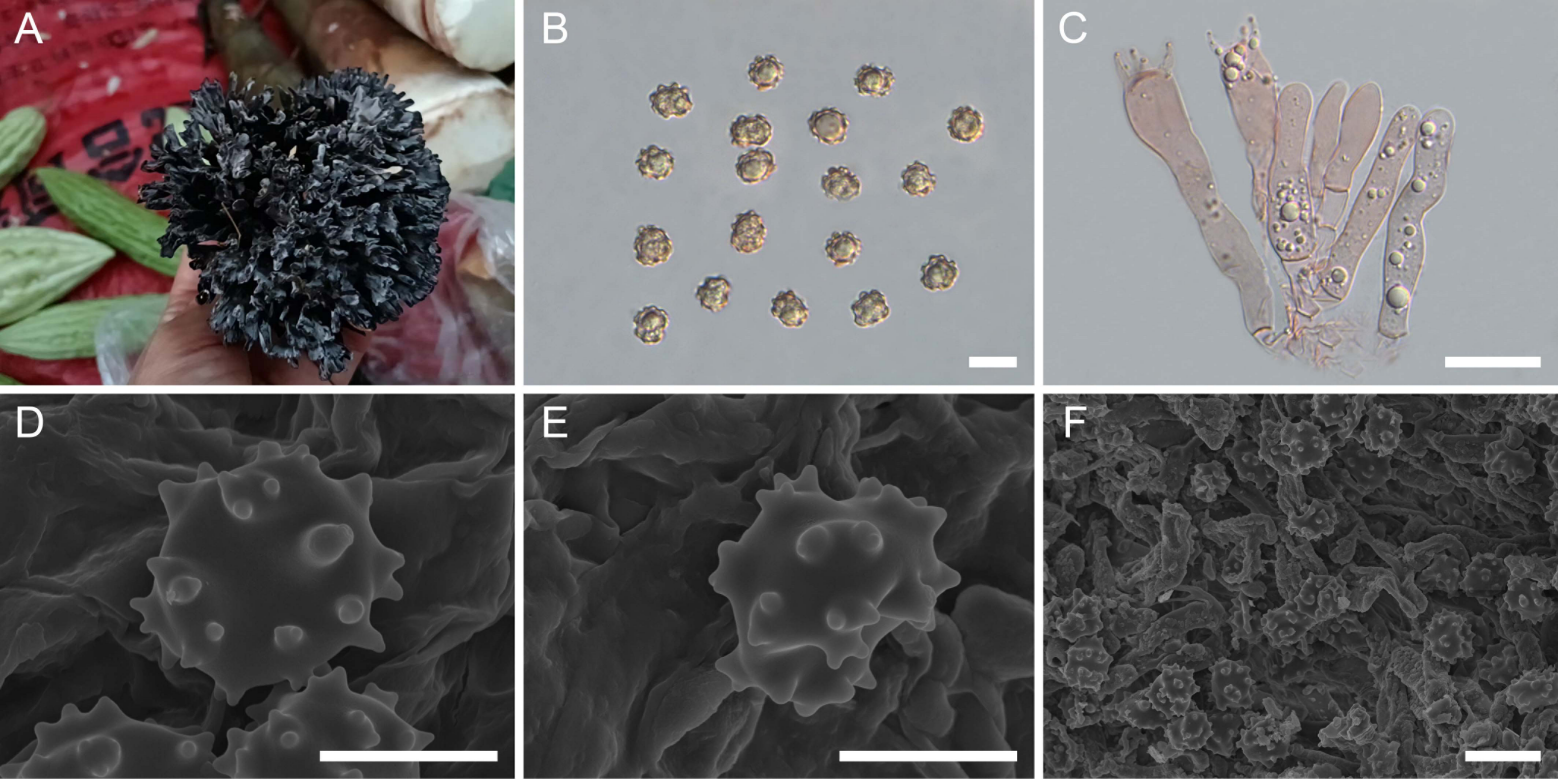
Macroscopic and microscopic features of *Thelephora densa* (MHKMU TJ Yu 293, holotype). **(A)** Basidiomata. **(B)** Basidiospores. **(C)** Basidia and basidioles. **(D–F)** Basidiospores under SEM. *Bars*, 10 µm **(B)**, 20 µm **(C)**, 5 µm **(D, E)**, 10 µm **(F)**.

**MycoBank:** 856474

**Etymology:** *densa* (Latin), refers to the dense branches of this species.

**Diagnosis:** Distinct from other species within the *T. palmata* complex in having relatively larger but thin basidiomata, with crowded and irregularly pleated branches, usually several adjacent branches forming a relatively wide complex at terminal, relatively wider basidiospores (8.5–10.1 µm × 8.1–9.5 µm) with short echinulis measuring 0.4–0.6 µm, and relatively longer basidia measuring 63.5–100 µm × 6–14.5 µm.

**Type:** China—Yunnan Province, Jingdong County, purchased from the local mushroom market, September 15, 2024, Taijie Yu 293 (MHKMU TJ Yu 293, holotype).

**Description:** Basidiomata solitary, scattered to gregarious, upright, dendritic, and merismatoid, in tufts 4–6 cm high and 4–7 cm broad, coriaceous and moist when fresh, while firm, brittle, and light in weight when dry; branches arising from a shared center or stipe, branched in three to four ranks, arranged in a spherical shape in top view, clavate to narrow spathulate or flabelliform initially, with age becoming more or less flattened, usually several adjacent branches forming a relatively wide complex at terminal, irregularly pleated, subglabrous, crowded, grayish-white (1C1), becoming grayish-black (2F1) when touched, 0.2–0.6 cm wide; terminal thin, 0.5–1 mm thick, rounded, entire or slightly lacerate forming short bifurcations or trifurcations. Hymenial surface grayish-white (1B1), becoming grayish-black (2F1) when touched, subglabrous, azonate. Context 0.1–0.4 cm thick, relatively thin at the margin and thick toward the base, concolourous with hymenial surface. Stipe 0.5–1 cm in length and 0.3–0.6 cm in diameter, irregularly conical to point-like at base, surface subglabrous, nearly black (1F3). Odor strong and agreeable upon drying. Taste not recorded.

Hymenium predominantly amphigenous. Basidiospores [60/2/2] 8.5–10.1 (–10.4) × (7.9–) 8.1–9.5 µm (ornamentations excluded), *Q* = 1.02–1.21 (–1.24), Qm = 1.10 ± 0.05, subglobose to broadly ellipsoid in frontal and lateral views, slightly thick-walled, inamyloid, brown in 5% KOH, and yellow-brown to brown in Melzer’s reagent; surface nodulose to verrucose, echinulis numerous and prominent, up to 0.4–0.6 µm high, usually in groups of two, occasionally isolated, then bifurcate in shape, subconical, but apex somewhat obtuse; hilar appendage oblique. Basidia 63.5–100 µm × 6–14.5 µm, clavate, four-spored, clamped at base, generally with many granulose contents; sterigmata 3–7.5 µm long. Basidioles numerous, similar to basidia in shape, but slightly smaller. Cystidia absent. Hyphal structure: Hyphal system monomitic, generative hyphae with clamp connections, and tissues turned to olive green in KOH. Hymenophoral trama composed of hyphae in a subparallel to an interwoven pattern; hyphae 3.5–7.5 µm wide, clamped, yellowish-brown, moderately branched, generally branched near clamp connections, occasionally flexuous and collapsed. Hair-like appendages of pointed margin composed of subparallel and loosely interwoven hyphae; hyphae cylindrical, thin-walled, clamped, occasionally branched, generally branched near clamp connections, 4–6 µm wide, terminal cell rounded at margin. Clamp connections abundant on hyphae of all tissues.

**Habitat:** Solitary, scattered to gregarious on the ground of tropical mixed broad-leaved forests dominated by *Castanea* spp. and *Quercus* spp.

**Additional specimens examined:** China—Yunnan Province, Jingdong County, purchased from the local mushroom market, September 15, 2024, Taijie Yu 295 (MHKMU TJ Yu 295).

**Known distribution:** Currently only known from Yunnan Province, China.

**Notes:** Macroscopically, *T. densa* from the tropics of China is recognized by its larger basidiomata, with more crowded branches, and wider basidiospores (8.5–10.1 µm × 8.1–9.5 µm) with short echinulis (measuring 0.4–0.6 µm). Morphologically, *T. densa* is reminiscent of three Chinese species (*T. apiculata*, *T. sinopalmata*, and *T. truncicola*) due to their similar basidiomata color. However, *T. apiculata* is distinguished by its sparser branches with conical and acute terminals and smaller basidiospores measuring 6.2–7.1 µm × 5.9–6.7 µm. *T. sinopalmata* is distinguished by its light-colored basidiomata, with whitish, sparser, subcylindrical to clavate papillate terminals and narrower basidiospores measuring 8.2–10.4 µm × 6.8–8.7 µm, with longer echinulis, up to 0.6–0.8 µm. *T. truncicola* is distinguished by its radially rugose or wrinkled basidiomata, sparser and shorter branches with whitish terminals, and narrower basidiospores measuring 8.3–10.2 µm × 6.7–8.5 µm, with longer echinulis, up to 0.9–1.3 µm.

Phylogenetically, *T. densa* formed a sister relationship with *T. esculenta*. Phylogenetically, *T. esculenta* formed a sister relationship with *T. densa*. Both of them are from the tropical forests of China and are commonly sold as edible mushrooms in the local mushroom market in Yunnan Province. They share very similar basidiospores and geographical distribution. However, *T. esculenta* differs in having larger basidiomata, with rounder, sparser, and thicker branches and shorter basidia measuring 50.5–64 µm × 8.5–11 µm.

***Thelephora esculenta*** L.P. Tang & T.J. Yu sp. nov.

(**Figures 5A–I**)

**Figure 5:**
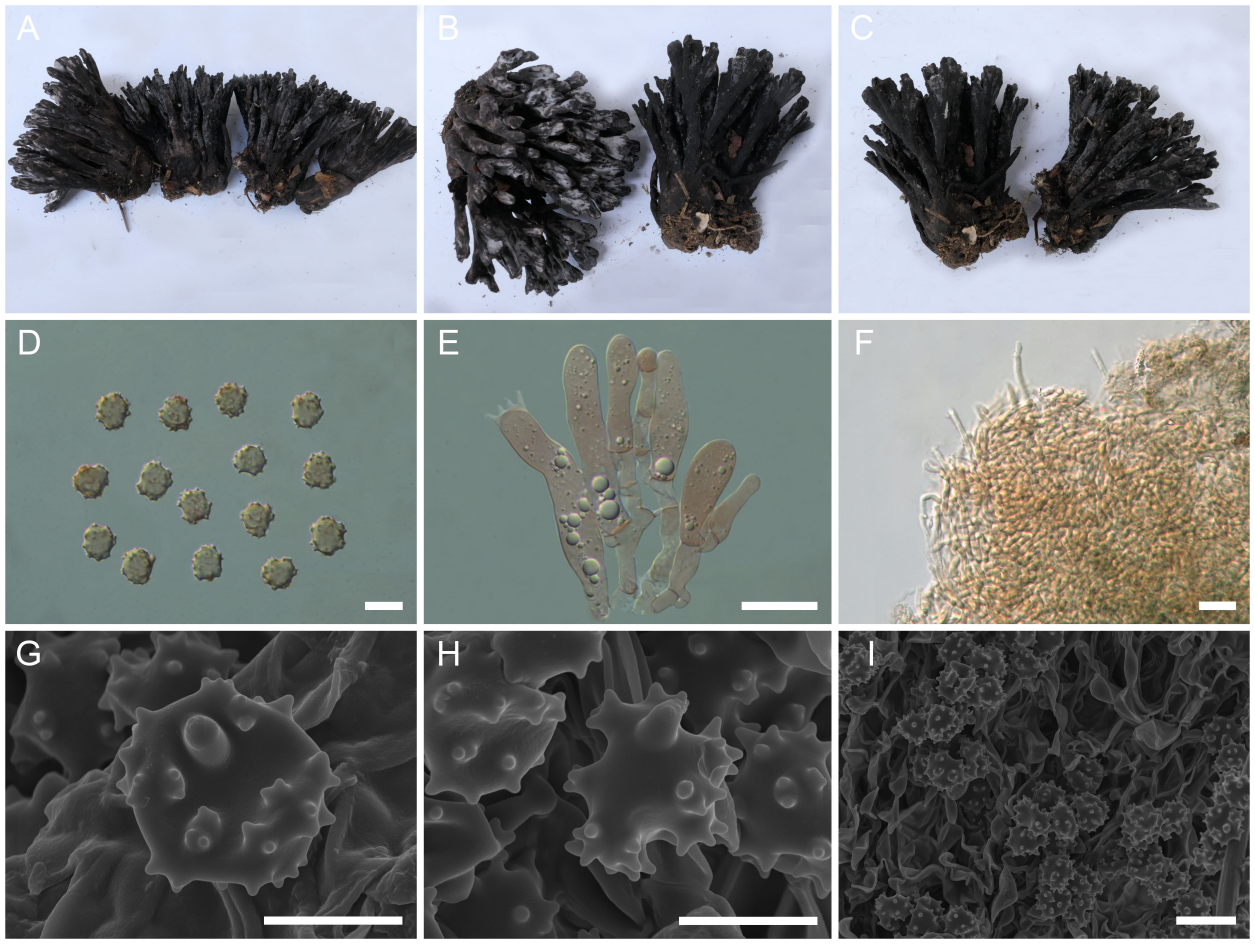
Macroscopic and microscopic features of *Thelephora esculenta* (MHKMU TJ Yu 243, holotype). **(A–C)** Basidiomata. **(D)** Basidiospores. **(E)** Basidia and basidioles. **(F)** Hair-like appendages of pointed tips. **(G–I)** Basidiospores under SEM. *Bars*, 10 µm **(D)**, 20 µm **(E)**, 25 µm **(F)**, 5 µm **(G, H)**, and 10 µm **(I)**.

**MycoBank:** 856468

**Etymology:** *esculenta* (Latin), refers to the new species being edible.

**Diagnosis:** Distinct from other species within the *T. palmata* complex in having relatively large and concrescent basidiomata, with dark grayish, subglabrous, thicker branches, becoming charcoal black when touched, relatively large basidiospores (8.6–10.2 µm × 7.6–9.5 µm) with short echinulis, up to 0.5–0.7 µm, and occurrence under trees of *Castanea* spp., *Quercus* spp., and *K. evelyniana* in tropical forests.

**Type:** China—Yunnan Province, Guangnan County, purchased from a local mushroom market, July 5, 2024, Taijie Yu 243 (MHKMU TJ Yu 243, holotype).

**Description:** Basidiomata gregarious, caespitose to concrescent, upright, dendritic, and merismatoid, in tufts 4–6 cm high and 5–8 cm broad, coriaceous and moist when fresh, while firm and light in weight when dry; branches arising from a shared center or stipe, branched in two to three ranks, subcylindrical and narrower spathulate initially, clavate to spathulate when mature, subglabrous, initially grayish-white (1C1), later dark gray (1D1), becoming charcoal black (1F3) when touched, 0.2–0.5 cm wide; terminal thin, 0.5–1 mm thick, rounded, entire or slightly lacerate forming bifurcations or trifurcations. Hymenial surface dark gray (1D1), becoming charcoal black (1F3) when touched, subglabrous, azonate. Context 0.3–0.6 cm thick, relatively thin at the margin and thick toward the base, concolourous with hymenial surface. Stipe 1–1.5 cm in length and 1.5–2 cm in diameter, irregularly conical to point-like at base, surface smooth to subglabrous, charcoal black (1F3). Basal hyphae grayish (1C1). Odor strong but agreeable, especially upon drying. Taste not recorded.

Hymenium predominantly amphigenous. Basidiospores [60/4/2] (8.3–) 8.6–10.2 (–10.7) × (7.2–) 7.6–9.5 µm (ornamentations excluded), *Q* = (1.01–) 1.02–1.22 (–1.25), Qm = 1.11 ± 0.06, subglobose to broadly ellipsoid in frontal and lateral views, slightly thick-walled, inamyloid, yellowish-brown to pale brown in 5% KOH, and yellow-brown to brown in Melzer’s reagent; surface nodulose to verrucose, echinulis numerous and prominent, up to 0.5–0.7 µm high, usually in groups of two, sometimes isolated, then bifurcate in shape, conical but apex, somewhat obtuse; hilar appendage oblique. Basidia (43.5–) 50.5–64 (–72) × (5.8–) 8.5–11 µm, clavate, four-spored, clamped at the base, generally with many granulose contents; sterigmata 3.3–7.5 µm long. Basidioles numerous, similar to basidia in shape, but slightly smaller. Cystidia absent. Hyphal structure: Hyphal system monomitic, generative hyphae with clamp connections, and tissues turned olive green in KOH. Hymenophoral trama composed of hyphae in a subparallel to an interwoven pattern; hyphae 3–5 µm wide, clamped, brownish, moderately branched, generally branched near clamp connections, occasionally flexuous, and collapsed. Hair-like appendages of pointed margin composed of subparallel and loosely interwoven hyphae; hyphae cylindrical, thin-walled, clamped, occasionally branched, and generally branched near clamp connections, 3–4 µm wide, terminal cell rounded at margin. Clamp connections abundant on hyphae of all tissues.

**Habitat:** Gregarious, caespitose to concrescent on the ground of tropical mixed coniferous–broad forests dominated by *Castanea* spp., *Quercus* spp., and *K. evelyniana*.

**Additional specimens examined:** China—Yunnan Province, Guangnan County, purchased from the local mushroom market, July 5, 2024, Taijie Yu 244 (MHKMU TJ Yu 244).

**Known distribution:** Currently known from Yunnan Province, China.

**Notes:** Morphologically, the *T. esculenta* from China is recognized by its relatively large and concrescent basidiomata, with dark grayish-black and thick branches and relatively large basidiospores (8.6–10.2 µm × 7.6–9.5 µm) with short echinulis, up to 0.5–0.7 µm. In terms of basidiomatal color, *T. esculenta* is reminiscent of two Chinese taxa, *T. apiculata* and *T. truncicola*. However, *T. apiculata* has a relatively smaller basidiomata, usually solitary, rarely concrescent, tomentose to velvety branches with conical and acute margin, and smaller basidiospores measuring 6.2–7.1 µm × 5.9–6.7 µm. *T. truncicola* is distinguished by its smaller and shorter basidiomata, gray-black to nearly black and thinner branches with radially rugose or wrinkled surface, whitish and flat terminals, and narrower basidiospores (8.3–10.2 µm× 6.7–8.5 µm), with longer echinulis measuring 0.9–1.3 μm.

Phylogenetically, *T. esculenta* formed a sister relationship with *T. densa*. For differences in these two taxa, see notes on *T. densa*.

***Thelephora fuscidula*** L.P. Tang & T.J. Yu sp. nov.

(**Figures 6A–I**)

**Figure 6:**
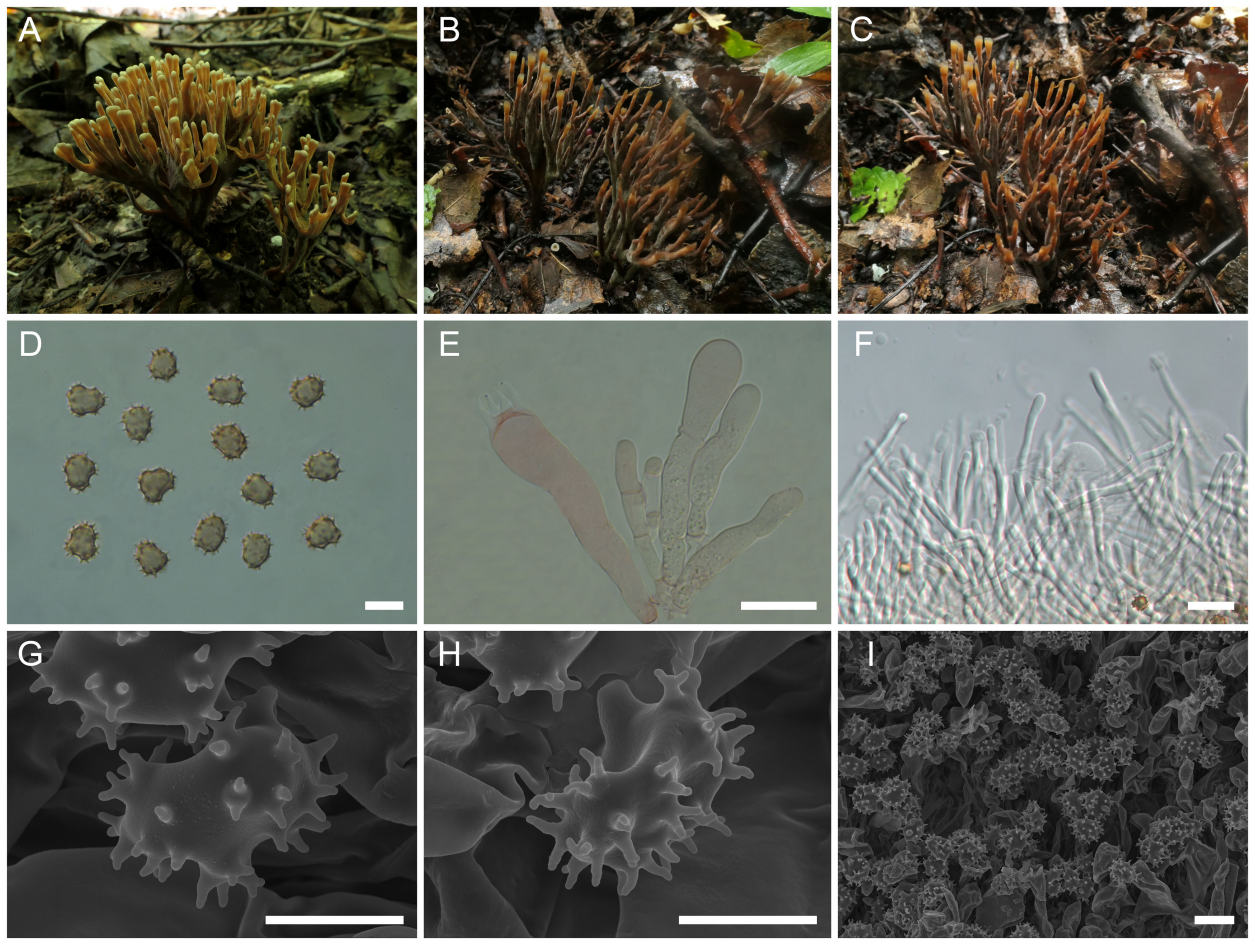
Macroscopic and microscopic features of *Thelephora fuscidula*. **(A–C)** Basidiomata: from MHKMU HY Huang 359, holotype **(A)**, and from MHKMU M Mu 357 **(B, C)**. **(D)** Basidiospores. **(E)** Basidia and basidioles. **(F)** Hair-like appendages of pointed tips. **(G–I)** Basidiospores under SEM. *Bars*, 10 µm **(D)**, 20 µm **(E)**, 25 µm **(F)**, 5 µm **(G, H)**, and 10 µm **(I)**.

**MycoBank:** 856469

**Etymology:** *fuscidula* (Latin), refers to the yellowish-brown terminals of the branches.

**Diagnosis:** Distinct from other species within the *T. palmata* complex in having yellowish-brown, glabrous, spathulate to narrow petaloid branches, sometimes radially rugulose or wrinkled, entire and oblate terminals, relatively long and significant stipe, short basidiospores (7.9–9.6 µm × 6.7–8 µm) with long echinulis, up to 0.9–1.5 µm, and occurrence under trees of *Betula platyphylla*, *Corylus mandshurica*, and *Q. mongolica* in temperate forests.

**Type:** China—Jilin Province, Antu County, Changbai Mountain, 42°24'03" N, 128°06'01" E, elevation 750 m, August 18, 2019, on the ground of mixed broad-leaved forests dominated by *B. platyphylla*, *C. mandshurica*, and *Q. mongolica*, Hongyan Huang 359 (MHKMU HY Huang 359, holotype).

**Description:** Basidiomata solitary to gregarious, upright, coralloid, and merismatoid, in tufts 4–5 cm high and 2–3 cm broad, coriaceous and moist when fresh, while firm, brittle, and light in weight when dry; branches arising from a shared stipe, branched in three to four ranks, subcylindrical initially, later flattened, narrow spathulate to narrow clavate when mature, 3–5 mm wide, sulcate; terminal thin, up to 1–2 mm thick, entire, round and flat. Hymenial surface glabrous, pale yellowish brown (2A3), yellowish-brown (2D5) to brown (3D4), radially rugulose or wrinkled, azonate. Context 3–4 mm thick, relatively thin at the margin and thick toward the base, brownish (2B1). Stipe grayish-brown (3D2) to grayish-black (2F1), 1.5–2 cm in length and 0.3–0.5 cm in diameter, irregularly flattened or slightly round at the base, surface smooth to slightly rugose. Odor mild when fresh, strong but agreeable upon drying. Taste not recorded.

Hymenium predominantly amphigenous. Basidiospores [60/3/2] (7.2–) 7.9–9.6 (–9.7) × (6.5–) 6.7–8 (–8.3) µm (ornamentations excluded), *Q* = (1.07–) 1.09–1.32 (–1.35), Qm = 1.20 ± 0.07, subglobose to broadly ellipsoid in frontal and lateral views, slightly thick-walled, inamyloid, yellowish-brown to brown in 5% KOH, and yellow-brown to brown in Melzer’s reagent; surface echinulate, echinulis numerous and prominent, up to 0.9–1.5 µm high, usually isolated or in groups of two, then bifurcate in shape, conical, but apex somewhat obtuse; hilar appendage oblique. Basidia (51–) 56–77 µm × 9.7–14.8 µm, clavate, four-spored, clamped at base, sometimes with finely granulose contents; sterigmata 5.3–9.7 µm long. Basidioles numerous, similar to basidia in shape, but slightly smaller. Cystidia absent. Hyphal structure: Hyphal system monomitic, generative hyphae with clamp connections. Hymenophoral trama composed of hyphae in a subparallel to an interwoven pattern; hyphae 3–7 µm wide, clamped, moderately branched, generally branched near clamp connections, occasionally flexuous and collapsed. Hair-like appendages of pointed margin composed of subparallel and loosely interwoven hyphae; hyphae cylindrical, thin-walled, clamped, occasionally branched, generally branched near clamp connections, 3.5–4.5 µm wide, terminal cell rounded at margin. Clamp connections abundant on hyphae of all tissues.

**Habitat:** Solitary to gregarious on the ground of temperate mixed coniferous–broad forests dominated by *B. platyphylla*, *C. mandshurica*, and *Q. mongolica*.

**Additional specimens examined:** China—Jilin Province, Antu County, Changbai Mountain, 42°21'11" N, 127°58'16" E, elevation 750 m, August 21, 2019, on the ground of mixed broad-leaved forests dominated by *B. platyphylla*, *C. mandshurica*, and *Q. mongolica*, Man Mu 357 (MHKMU M Mu 357).

**Known distribution:** Currently known from China (Jilin Province) and Sweden.

**Notes:** Morphologically, the *T. fuscidula* from the temperate regions of Eurasia is recognized by its yellowish-brown and almost smooth branches with entire terminals, relatively long and significant stipe, and short basidiospores (7.9–9.6 µm × 6.7–8 µm) with long echinulis measuring 0.9–1.5 µm. In terms of basidiomatal colors, *T. fuscidula* resembles *T. pinnatifida*. However, *T. pinnatifida*, so far restricted to southwestern China, has velvety, clavate to pinnatifid or ramiform branches, with chalky white to orange white, deeply lacerate terminals, small basidiospores with shorter echinulis measuring 0.3–0.6 µm, and association with Fagaceae and Pinaceae (Tian et al., 2024). In addition, both species differ in the shape of their basidiomata, with the basidiomata of *T. fuscidula* being upright, whereas those of *T. pinnatifida* are rosette-like fans lying low on the forest floor looking as though they have been trodden on (Tian et al., 2024).

Phylogenetically, *T. fuscidula* formed a sister relationship with “*Thelephora palmata*”-1 from North America (USA), and both of them have a temperate distribution. Interestingly, a sequence (AF272919), labeled as “*T. palmata*” from Sweden, was clustered with this new species, indicating *T. fuscidula* having a wide distribution in temperate areas of Asia and Europe.

***Thelephora sinopalmata*** L.P. Tang & T.J. Yu sp. nov.

(**Figures 7A–I**)

**Figure 7:**
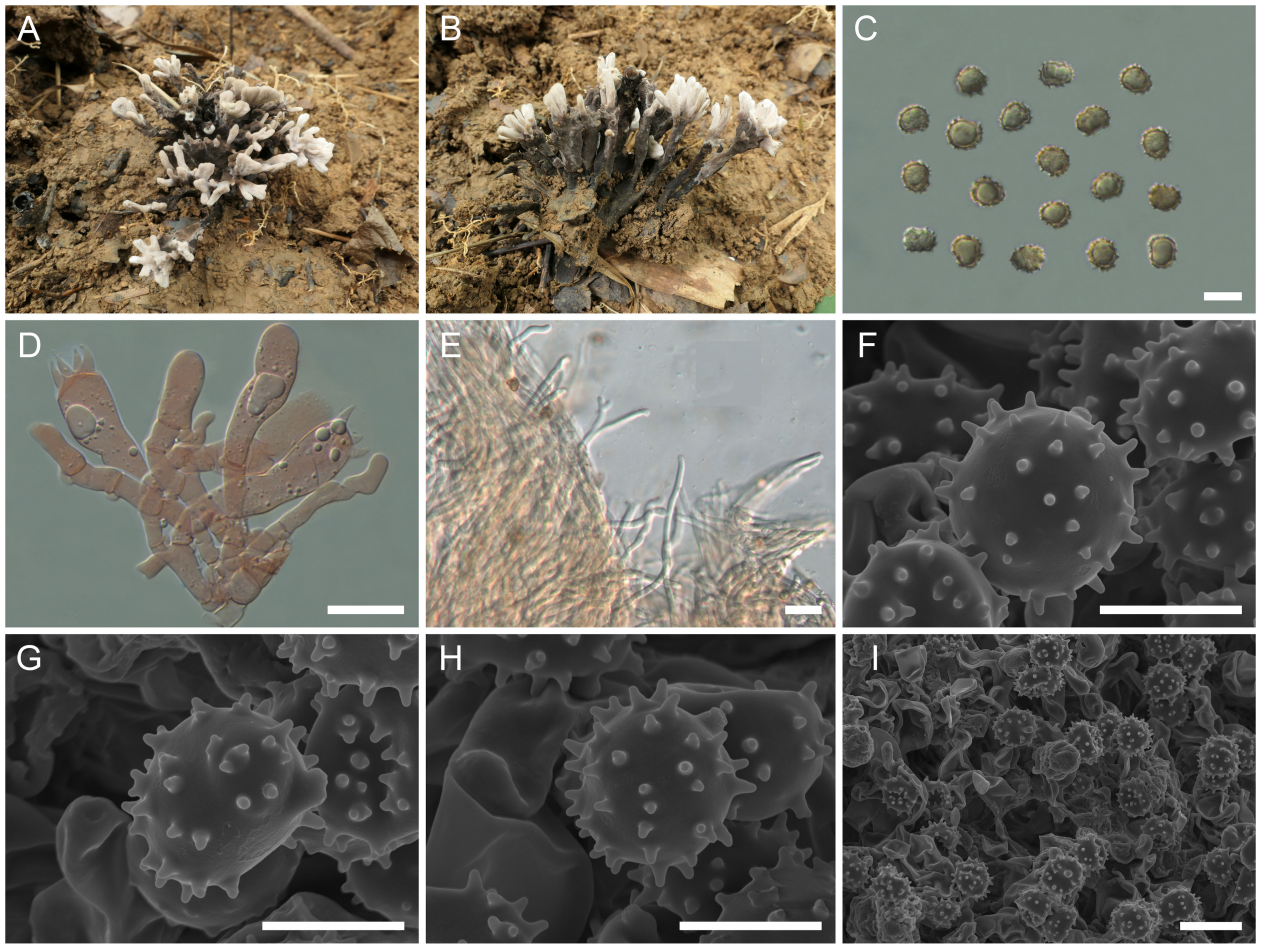
Macroscopic and microscopic features of *Thelephora sinopalmat*a (MHKMU R Xue 190, holotype). **(A, B)** Basidiomata. **(C)** Basidiospores. **(D)** Basidia and basidioles. **(E)** Hair-like appendages of pointed tips. **(F–I)** Basidiospores under SEM. *Bars*, 10 µm **(C)**, 20 µm **(D)**, 25 µm **(E)**, 5 µm **(F–H)**, and 10 µm **(I)**.

**MycoBank:** 856472

**Etymology:** *sinopalmata* (Latin), refers to the similarity of the new species to *T. palmata* and is found in China.

**Diagnosis:** Distinct from other species within the *T. palmata* complex in having dark gray, smooth to papillate, subcylindrical to clavate branches, forming a petal shape, with whitish, entire, blunt, and round terminals, short to insignificant stipe, relatively large basidiospores (8.2–10.4 µm × 6.8–8.7 µm) with medium-length echinulis measuring 0.6–0.8 µm, and occurrence under Fagaceae trees in subtropical forests.

**Type:** China—Anhui Province, Shitai County, Qidu Town, Jinzhu Mountain, 30°16'01" N, 117°47'08" E, elevation 500 m, July 16, 2024, on the ground of broad-leaved forests dominated by *C. seguinii*, *Phyllostachys heterocycla*, and *Quercus* spp., Rou Xue 190 (MHKMU R Xue 190, holotype).

**Description:** Basidiomata solitary, gregarious, upright, dendritic, and merismatoid, in tufts 5 cm high and 5 cm broad, coriaceous and moist when fresh, while corky and light in weight when dry; branches arising from a shared center or stipe, branched in two to three ranks, subcylindrical to clavate, smooth to papillate, 0.4–0.8 cm wide; terminal thin, 0.5–0.8 mm thick, subglabrous to subglabrous, flat, whitish (1A2) to pale grayish-white (4D3), forming a petal shape. Hymenial surface subglabrous, velvety, whitish (1A2) initially, darkening to gray (1E1), dark gray (1E1) when fully mature, azonate. Context 1–3 mm thick, relatively thin at the margin and thick toward the base, gray brown (4D2). Stipe concolourous with hymenial surface or slightly darker, (0–) 0.2–0.8 cm in length and (0–) 0.2–0.4 cm in diameter, irregularly subcylindrical to flattened or broadened at the base, surface subglabrous, slightly rugose. Odor mild and slightly fragrant upon drying. Taste not recorded.

Hymenium predominantly amphigenous. Basidiospores [80/1/1] (7.5–) 8.2–10.4 (–10.8) × (6.5–) 6.8–8.7 (–8.9) µm (ornamentations excluded), *Q* = (1.01–) 1.04–1.31 (–1.37), Qm = 1.16 ± 0.08, subglobose to broadly ellipsoid in frontal and lateral views, echinulate, irregular in outline, slightly thick-walled, inamyloid, yellow-brown to brownish in 5% KOH, and brown in Melzer’s reagent; surface echinulate, echinulis numerous and prominent, up to 0.6–0.8 µm high, usually isolated, occasionally in groups of two, then bifurcate in shape, subconical, but apex somewhat obtuse; hilar appendage oblique. Basidia 51.5–77.5 (–83) µm × 11.5–16.5 µm, clavate, four-spored, clamped at base, occasionally with finely granulose contents; sterigmata 6–7.8 µm long. Basidioles numerous, similar to basidia in shape, but slightly smaller. Cystidia absent. Hyphal structure: Hyphal system monomitic, generative hyphae with clamp connections, and tissues turned olive green in KOH. Hymenophoral trama composed of hyphae in a subparallel to an interwoven pattern; hyphae 4–6.5 µm wide, clamped, moderately branched, generally branched near clamp connections, occasionally flexuous and collapsed. Hair-like appendages of pointed tips composed of subparallel and loosely interwoven hyphae; hyphae cylindrical, thin-walled, clamped, occasionally branched, generally branched near clamp connections, 3.5–5 µm wide, terminal cell rounded at tips, rarely inflating, up to 12 µm wide. Clamp connections abundant on hyphae of all tissues.

**Habitat:** Solitary to gregarious on the ground of subtropical mixed broad-leaved forests dominated by *C. seguinii* and *Quercus* spp.

**Known distribution:** Currently known from Anhui Province, China.

**Notes:** Macroscopically, the *T. sinopalmata* from the subtropical regions of China is recognized by its dark gray and smooth to papillate branches, with whitish entire and subglabrous terminals, short to insignificant stipe, and relatively large basidiospores (8.2–10.4 µm × 6.8–8.7 µm) with medium-length echinulis measuring 0.6–0.8 µm. Morphologically, *T. sinopalmata* closely resembles *T. apiculat*a and *T. palmata*. Of them, *T. apiculata* has a geographically overlapping distribution with *T. sinopalmata* in Anhui Province. However, *T. apiculata* is distinguished in having larger branches, with darker conical and acute terminals and small basidiospores measuring 6.2–7.1 µm × 5.9–6.7 µm. The *T. palmata* from the temperate regions of Europe is distinguished by its fuscous purple, chocolate brown to blackish-brown basidiomata, often with a violet tone at maturity, relatively larger basidiospores measuring 8–12 µm × 6–8 µm, rather fetid flavor, particularly upon drying, and its association with conifers (Fries, 1821; Coker, 1921).

Phylogenetically, *T. sinopalmata* had no sister to be confirmed in the present study, but it formed a moderately supported (BS = 72, PP = 0.99) clade comprising three Chinese species (*T. cornu-damae*, *T. esculenta*, and *T. densa*), three North American phylogenetic species (“*T. palmata*”-1, “*T. palmata*”-2, and “*T. palmata*”-3), and one Eurasian species (*T. fuscidula*) and is located at the base of the clade. Among these, *T. cornu-damae* is distinguished by its pale grayish basidiomata, with spathulate to narrow flabelliform branches, deeply lacerate and serrulate terminals, longer and significant stipe, and basidiospores with longer echinulis, up to 1.2–1.5 µm. *T. esculenta* is distinguished by its larger and thicker basidiomata, with darker branches becoming charcoal black when touched. *T. fuscidula*, distributed in temperate regions of Eurasia, has yellowish-brown and flatted terminals, relatively long and significant stipe, and smaller basidiospores (7.9–9.6 µm × 6.7–8 µm) with longer echinulis measuring 0.9–1.5 µm. *T. densa*, restricted to tropical regions in southwestern China, has a spherical-shaped basidiomata, with more crowded branches, and longer basidiospores (8.5–10.1 µm × 8.1–9.5 µm) with shorter echinulis measuring 0.4–0.6 µm.

***Thelephora truncicola*** L.P. Tang & T.J. Yu sp. nov.

(**Figures 8A–I**)

**Figure 8:**
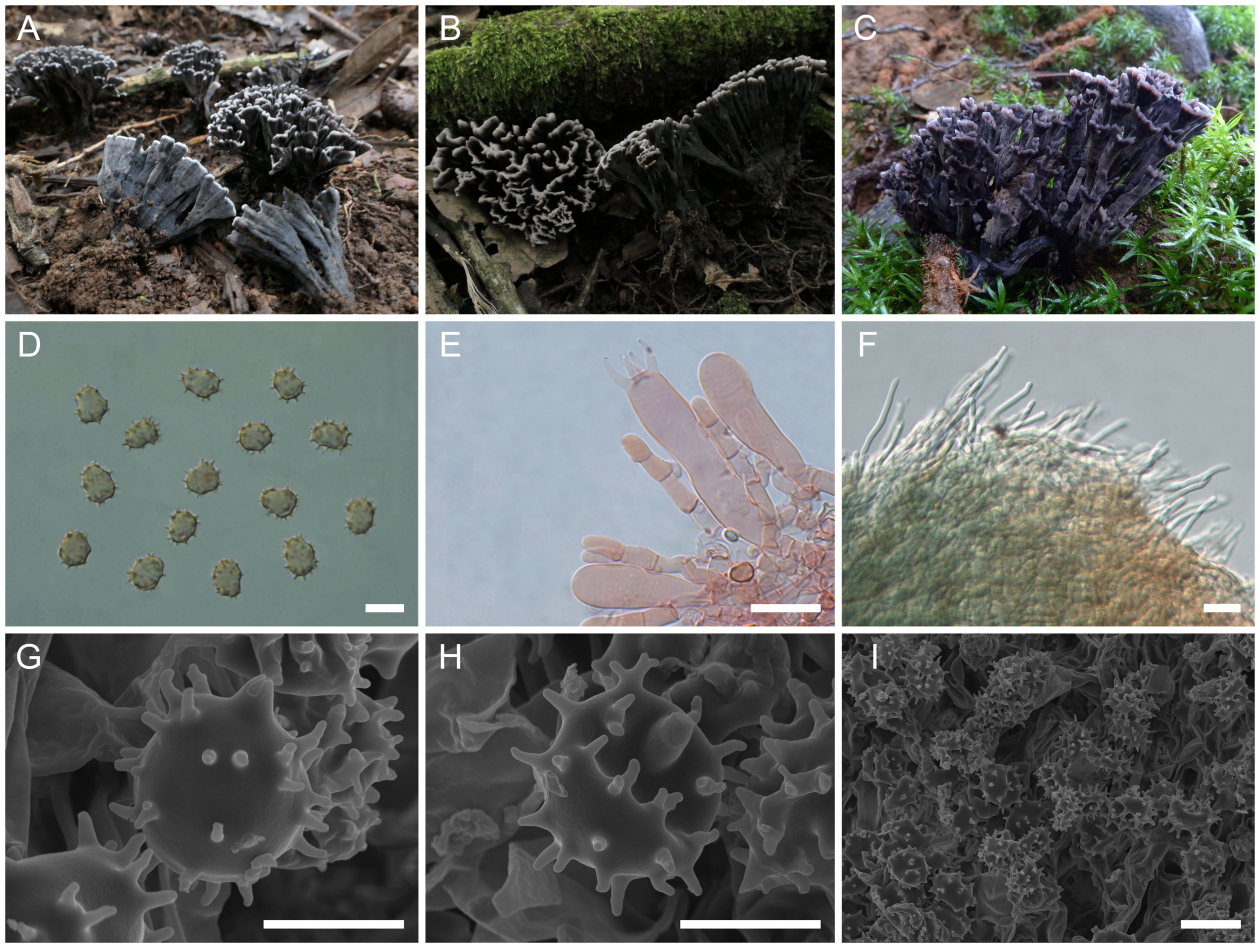
Macroscopic and microscopic features of *Thelephora truncicola*. **(A–C)** Basidiomata: from MHKMU YJ Pu 097, holotype **(A)**; from MHKMU T Huang 224-1 **(B)**; and from MHKMU LP Tang 2565 **(C)**. **(D)** Basidiospores. **(E)** Basidia and basidioles. **(F)** Hair-like appendages of pointed tips. **(G–I)** Basidiospores under SEM. *Bars*, 10 µm **(D)**, 20 µm **(E)**, 25 µm **(F)**, 5 µm **(G, H)**, and 10 µm **(I)**.

**MycoBank:** 856471

**Etymology:** *truncicola* (Latin), refers to the truncated and flat terminals of the branches.

**Diagnosis:** Distinct from other species within the *T. palmata* complex in having small and short basidiomata, with gray-black to nearly black wide spathulate to flabelliform, radially rugulose or wrinkled branches, pale whitish and short terminals, short and inconspicuous stipe, relatively large basidiospores (8.3–10.2 µm × 6.7–8.5 µm) with long echinulis measuring 0.9–1.3 µm, and occurrence under trees of *Quercus* spp. in subtropical forests.

**Type:** China—Yunnan Province, Shizong County, Junzi Mountain, 24°38'01" N, 104°09'36" E, elevation 2,300 m, August 10, 2019, on the ground of mixed broad-leaved forests dominated by *Quercus* spp. and a few *Rhododendron* spp., Yunju Pu 097 (MHKMU YJ Pu 097, holotype).

**Description:** Basidiomata caespitose to gregarious, upright, dendritic, and merismatoid, in tufts 2–4 cm high and 3–5 cm broad, coriaceous and more or less moist when fresh, while firm, brittle, and light in weight when dry; branches arising from a shared center or stipe, branched in two to three ranks, clavate to narrow spathulate when young, while wide spathulate to flabelliform when mature, visibly ribbed; terminal thin, 1–2 mm thick, entire, short, flat, and pale grayish-white (2A1). Hymenial surface subglabrous, grayish-black (1F1) to nearly black (4F1), nearly smooth to somewhat radially rugulose or wrinkled, azonate. Context 2–4 mm thick, relatively thin at the margin and thick toward the base, white (1A1), gray-white (3E1). Stipe 0.5–1 cm in length and 0.4–0.6 cm in diameter, irregularly cylindrical to flattened or broadened at the base, surface smooth to slightly rugose, dark gray (3F1) to gray-black (1F1). Odor strong and slightly foul when fresh, especially upon drying. Taste not recorded.

Hymenium predominantly amphigenous. Basidiospores [80/4/3] (8.2–) 8.3–10.2 (–10.7) × (6.5–) 6.7–8.5 (–9) µm (ornamentations excluded), *Q* = (1.03–) 1.12–1.37 (–1.39), Qm = 1.24 ± 0.07, subglobose to broadly ellipsoid in frontal and lateral views, slightly thick-walled, inamyloid, yellowish-brown to pale brown in 5% KOH, and yellow brown to brown in Melzer’s reagent; surface echinulate, echinulis numerous and prominent, up to 0.9–1.3 µm high, usually isolated or in groups of two, then bifurcate in shape, conical, but apex somewhat obtuse; hilar appendage oblique. Basidia 48.5–68.5 (–74) × 8.5–13 µm, clavate, four-spored, clamped at the base, occasionally with finely granulose contents; sterigmata 6.8–9.5 µm long. Basidioles numerous, similar to basidia in shape, but slightly smaller. Cystidia absent. Hyphal structure: Hyphal system monomitic, generative hyphae with clamp connections, and tissues turned olive green in KOH. Hymenophoral trama composed of hyphae in a subparallel to an interwoven pattern; hyphae 3.5–6 µm wide, clamped, moderately branched, generally branched near clamp connections, occasionally flexuous and collapsed. Hair-like appendages of pointed margin composed of subparallel and loosely interwoven hyphae; hyphae cylindrical, thin-walled, clamped, occasionally branched, generally branched near clamp connections, 3.5–5.5 µm wide, and terminal cell rounded at margin. Clamp connections abundant on hyphae of all tissues.

**Habitat:** Caespitose to gregarious on the ground of subtropical mixed broad-leaved forests dominated by *Quercus* spp. and a few Ericaceae.

**Additional specimens examined:** China—Yunnan Province, Shizong County, Junzi Mountain, 24°38'44" N, 104°09'20" E, elevation 2,300 m, July 23, 2020, on the ground dominated by *Quercus* spp. in mixed broad-leaved forests, Ting Huang 224-1 (MHKMU T Huang 224-1); same location, 24°31'01" N, 104°09'51" E, elevation 2,250 m, August 23, 2018, on the ground dominated by *Quercus* spp. and a few Ericaceae in mixed broad-leaved forests, Liping Tang 2565 (MHKMU LP Tang 2565).

**Known distribution:** Currently known from Yunnan Province, China.

**Notes:** Morphologically, the *T. truncicola* from the subtropical regions of China is recognized by its shorter basidiomata, with gray-black to nearly black branches, whitish terminals, short to inconspicuous stipe, and relatively large basidiospores (8.3–10.2 µm × 6.7–8.5 µm) with long echinulis measuring 0.9–1.3 µm. *T. truncicola* is easy to recognize at maturity and not often confused with other species, except for *Thelephora aquila* S.R. Yang, Y.L. Wei & H.S. Yuan. However, *T. aquila* is distinguished by its flabelliform to applanate-lobate basidiomata, with a strongly lobed and wavy margin (Yang et al., 2023). In terms of color, *T. truncicola* is similar to *T. dactyliophora*, *T. palmata*, and *T. pinnatifida* when not mature. However, *T. dactyliophora* differs in having spathulate to narrow petaloid basidiomata, with gray to brownish-gray branches, orange-white and deeply lacerate terminals, and smaller basidiospores measuring 5–7.5 μm × 4–6.5 μm, with shorter echinulis (0.3–0.5 μm). *T. pinnatifida* has a geographically overlapping distribution with *T. truncicola* in Yunnan Province, but the former differs in having brownish-orange to brown branches, with needle-like and orange-white terminals, and a yeast powder flavor when dried (Tian et al., 2024).

Phylogenetically, *T. truncicola* had no sister to be confirmed in the present study, but it formed a moderately supported (BS = 100, PP = 1) clade comprising four Chinese species (*T. cornu-damae*, *T. esculenta*, *T. sinopalmata*, and *T. densa*), three North American phylogenetic species (“*T. palmata*”-1, “*T. palmata*”-2, and “*T. palmata*”-3), and one Eurasian species (*T. fuscidula*) and is located at the base of the clade.

***Thelephora truncicola* f. *pallescens*** L.P. Tang & T.J. Yu sp. nov.

(**Figures 9A–I**)

**Figure 9:**
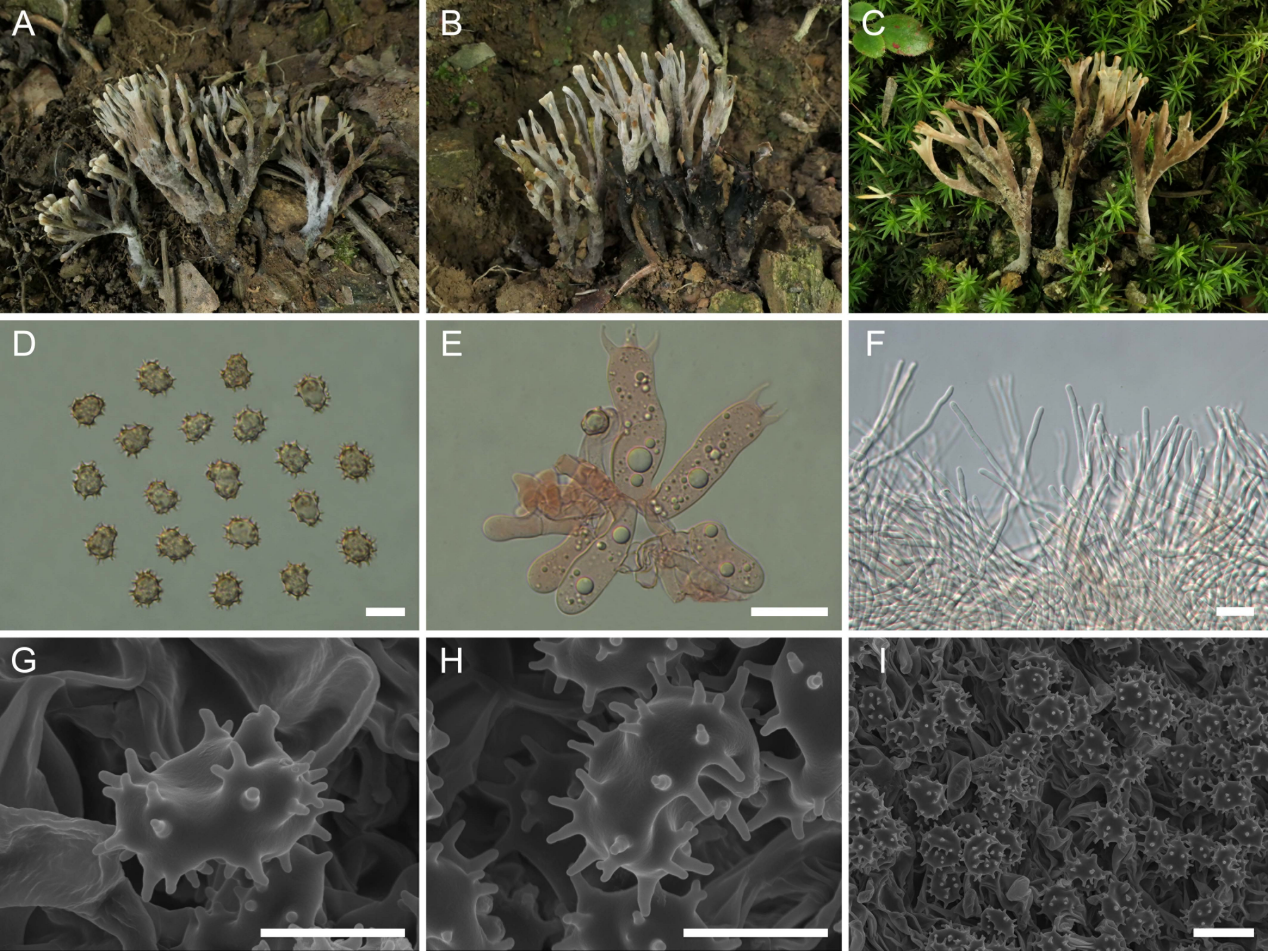
Macroscopic and microscopic features of *Thelephora truncicola* f. *pallescens*. **(A–C)** Basidiomata: from MHKMU TJ Yu 255, holotype **(A, B)**, and from MHKMU LJ Su 408 **(C)**. **(D)** Basidiospores. **(E)** Basidia and basidioles. **(F)** Hair-like appendages of pointed tips. **(G–I)** Basidiospores under SEM. *Bars*, 10 μm **(D)**, 20 μm **(E)**, 25 μm **(F)**, 5 μm **(G, H)**, and 10 μm **(I)**.

**MycoBank:** 856470

**Etymology:** *pallescens* (Latin), refers to the light-colored branches of this species compared to *T. truncicola*.

**Diagnosis:** Distinct from *T. truncicola* in having pale gray and slightly tomentose basidiomata, with pale gray to pale grayish-brown and longer branches, pale yellowish-gray and narrower terminals, lacerate to entire, forming bifurcate, longer and significant stipe, and occurrence under *Lithocarpus* spp. trees in subtropical forests.

**Type:** China—Anhui Province, Shitai County, Renli Town, Shanshan Forest Park, 30°16'58" N, 117°32'44" E, elevation 780 m, July 19, 2024, on the ground dominated by *Lithocarpus henryi* and a few *Camellia* spp., *C. mollissima*, and *Quercus* spp. in mixed broad-leaved forests, Taijie Yu 255 (MHKMU TJ Yu 255, holotype).

**Description:** Basidiomata solitary, scattered to gregarious, upright, coralloid, and merismatoid, in tufts 3–5 cm high and 3–4 cm broad, coriaceous and moist when fresh, while firm, brittle, and light in weight when dry; branches arising from a shared stipe, branched in two to four ranks, flattened, clavate to narrow spathulate initially, spathulate to narrow flabelliform or wide petaloid when mature, subglabrous, 0.3–0.5 cm wide; terminal thin, 0.4–0.6 mm thick, moderately lacerate and serrulate, forming a bifurcate to trifurcate shape, pale gray (1C1) to pale grayish-white (1B2). Hymenial surface subglabrous, azonate, surface with powdery adherence, grayish-white (2B1), pale grayish (2C1) to pale grayish-brown (2C2). Context 2–4 mm thick, relatively thin at the margin and thick toward the base, gray (2D1) to grayish-black (2E1). Stipe 1–2 cm in length and 0.2–0.7 cm in diameter, subcylindrical or slightly broadened at the base, surface nearly smooth, with powdery adherence, concolourous with branches or slightly darker. Odor mild when fresh, strawy flavor upon drying. Taste not recorded.

Hymenium predominantly amphigenous. Basidiospores [60/3/3] (7.8–) 8.2–10.2 (–10.5) × 6.5–8.8 (–9.5) µm (ornamentations excluded), *Q* = (1.01–) 1.03–1.33 (–1.42), Qm = 1.19 ± 0.09, subglobose to broadly ellipsoid in frontal and lateral views, slightly thick-walled, inamyloid, brown in 5% KOH, and yellow-brown to brown in Melzer’s reagent; surface echinulate, echinulis numerous and prominent, up to 1–1.5 µm high, usually isolated or in groups of two, then bifurcate in shape, conical, but apex somewhat obtuse; hilar appendage oblique. Basidia 42.5–59 µm× 11.5–14.7 µm, clavate, four-spored, clamped at the base, usually with numerous granulose contents; sterigmata 5–8.2 µm long. Basidioles numerous, similar to basidia in shape, but slightly smaller. Cystidia absent. Hyphal structure: Hyphal system monomitic, generative hyphae with clamp connections. Hymenophoral trama composed of hyphae in a subparallel to an interwoven pattern; hyphae 3–5 µm wide, clamped, moderately branched, generally branched near clamp connections, occasionally flexuous and collapsed. Hair-like appendages of pointed margin composed of subparallel and loosely interwoven hyphae; hyphae cylindrical, thin-walled, clamped, occasionally branched, generally branched near clamp connections, 3–4.5 µm wide, terminal cell rounded at margin. Clamp connections abundant on hyphae of all tissues.

**Habitat:** Solitary, scattered to gregarious on the ground of subtropical mixed broad-leaved forests dominated by *L. henryi* and a few *Castanea* spp. and *Quercus* spp.

**Additional specimens examined:** China—Anhui Province, Shitai County, Xianyu Town, Qifeng Village, Xianyu Scenic Area, 30°03'42" N, 117°17'23" E, elevation 200 m, July 27, 2022, on the ground dominated by *L. henryi* and a few *Quercus* spp. in mixed broad-leaved forests, Qing Deng 31 (MHKMU Q Deng 31). Shitai County, Jitan Town, near Hongdun Village, 30°15'34" N, 117°24'43" E, elevation 70 m, July 19, 2024, on the ground dominated by *L. henryi* and a few *C. mollissima* and *Quercus* spp. in mixed broad-leaved forests, Linjie Su 408 (MHKMU LJ Su 408).

**Known distribution:** Currently known from Anhui Province, China.

**Notes:** Macroscopically, the *T. truncicola* f. *pallescens* from the subtropical regions of China is recognized by its pale gray and slightly tomentose basidiomata, with grayish to pale grayish-brown branches and terminals, longer and significant stipe, and large basidiospores (8.2–10.2 µm × 6.5–8.8 µm) with long echinulis measuring 1–1.5 µm. *T. truncicola* f. *pallescens* is reminiscent of *T. cornu-damae* and *T. sinopalmata*, having light-colored basidiomata. However, *T. cornu-damae*, associated with *P. yunnanensis*, is distributed in high elevations of Yunnan Province and has wider and flat branches, and deeply lacerate and serrulate terminals. *T. sinopalmata* has a geographic overlapping distribution with *T. truncicola* f. *pallescens* in Anhui Province, but *T. sinopalmata* is distinguished by its whitish and thinner terminals, short to insignificant stipe, and shorter echinulis measuring 0.6–0.8 µm. Furthermore, the two species differ in host preferences. *T. sinopalmata* appears to prefer associating with *C. seguinii* and *Quercus* spp., while *T. truncicola* f. *pallescens* prefers associating with *Lithocarpus* spp.

## 4 Discussion

In the present research, we constructed a four-locus phylogenetic tree (ITS + nrLSU + *rpb2* + nrSSU) and demonstrated that what once was considered a single species, *T. palmata*, actually represents a species complex consisting of at least 12 unknown species. Before new names could be assigned to these undescribed species, the identification of the old name proved to be a challenge, particularly as it has been used for more than 200 years and its original description was not sufficiently detailed (Fries, 1821).

According to our phylogenetic tree, seven new taxa (i.e., *T. apiculata*, *T. cornu-damae*, *T. densa*, *T. esculenta*, *T. sinopalmata*, *T. truncicola*, and *T. truncicola* f. *pallescens*) from China and one new taxon (*T. fuscidul*a) from Eurasia are proposed, supported by diagnosable criteria including morphological characteristics, host preferences, and geographical distribution. With the exception of the mentioned new species, there is unrecognized cryptic diversity within the *T. palmata* complex from Asia, Europe, and North America. The remaining sequences, labeled as “*T. palmata*”, form five independent lineages, and the difference of the base pairs in the ITS regions is more than 1.5% nucleotides among these taxa; hence, these taxa should represent five different species and are provisionally treated as “*T. palmata*”-1 and “*T. palmata*”-3 from North America, “*T. palmata*”-2 from Europe, “*T. palmata*”-4 from Japan, and “*T. palmata*”-5 from Indonesia. Unfortunately, the precise clade representing the true *T. palmata* lineage needs to be elucidated until the acquisition of collections that correspond to the geographical and ecological origins of the species described by Fries (1821).

The traditional morphological concept of *T. palmata* agrees with our observations of the species complex. For the identification of species within the *T. palmata* complex, the following characteristics are quite helpful: microscopic characteristics, host preferences, and geographical distribution (see **Table 2** for details). Firstly, SEM characteristics are helpful to distinguish the complex species of *T. palmata*. For instance, the echinulis of basidiospores are significantly different within the *T. palmata* complex. Three species with similar macro-morphology, i.e., *T. apiculata*, *T. cornu-damae*, and *T. sinopalmata*, differ significantly in the shape and size of the basidiospores’ echinulis. Among them, *T. apiculata* has short echinulis measuring 0.3–0.5 µm, usually isolated or in groups of two; *T. cornu-damae* has long echinulis measuring 1.2–1.5 µm, usually in groups of two; and *T. sinopalmata* has medium-length and usually isolated echinulis measuring 0.6–0.8 µm.

**Table 2:** Diagnostic morphological characteristics of the species within the *Thelephora palmata* complex.

Taxa	Basidiospores (µm)	Echinulis (µm)	Basidia (µm)	Distribution	Hosts
*Thelephora apiculata*	6.2–7.1 × 5.9–6.7	0.3–0.5	46–62.8 × 7–10.5	Asia (E and SW China)	*Castanea*, *Quercus*
*T. cornu-damae*	8.6–10 × 7–8.4	1.2–1.5	62–74.5 × 9.5–14.5	Asia (SW China)	*Pinus yunnanensis*
*T. densa*	8.5–10.1 × 8.1–9.5	0.4–0.6	63.5–100 × 6–14.5	Asia (SW China)	*Castanea*, *Quercus*
*T. esculenta*	8.6–10.2 × 7.6–9.5	0.5–0.7	50.5–64 × 8.5–11	Asia (SW China)	*Castanea*, *Keteleeria*, *Quercus*
*T. fuscidula*	7.9–9.6 × 6.7–8	0.9–1.5	56–77 × 9.7–14.8	Asia (NE China) and Europe (Sweden)	*Betula*, *Corylus*, *Quercus*
*T. palmata*	8–12 × 7–9	**–**	70–100 × 9–12	Europe	Conifers
*T. sinopalmata*	8.2–10.4 × 6.8–8.7	0.6–0.8	51.5–77.5 × 11.5–16.5	Asia (E China)	Fagaceae
*T. truncicola*	8.3–10.2 × 6.7–8.5	0.9–1.3	48.5–68.5 × 8.5–13	Asia (SW China)	*Quercus*
*T. truncicola* f. *pallescens*	8.2–10.2 × 6.5–8.8	1–1.5	42.5–59 × 11.5–14.7	Asia (E China)	*Lithocarpus*

*E*, eastern; *NE*, northeastern; *SW*, southwestern

Secondly, the host preference and geographical distribution are quite helpful within the *T. palmata* complex. Indeed, the vast majority of species from China prefer broad-leaved trees (except for *T. cornu-damae*), predominantly Fagaceae, rather than the conifers described in the literature. Furthermore, species within this species complex have strict distribution patterns. Some phenetically and phylogenetically related species can be well distinguished by their host preferences and geographical distribution. For instance, three Chinese species—*T. cornu-damae*, *T. truncicola*, and *T. truncicola* f. *pallescens*—have similar morphological features and close phylogenetic affiliation. However, *T. cornu-damae* is restricted to southwestern China at high elevations (>2,000 m) and prefers *P. yunnanensis*, *T. truncicola* prefers *Quercus* spp. and is restricted to southwestern China at high elevations (>2,000 m), and *T. truncicola* f. *pallescens* prefers *Lithocarpus* spp. and is restricted to eastern China at low elevations (<800 m).

In addition, odor is often overlooked but is quite a useful characteristic within this species complex. For instance, *T. palmata* is known for its foul odor, particularly on drying (Fries, 1821; Coker, 1921), while the odor of *T. apiculata* is mild when fresh and is slightly sweet and fragrant after drying. The odor of *T. truncicola* f. *pallescens* is strawy after drying. It should be noted, however, that the recording of odor is not only dependent on the subjective feeling of the collector but is also difficult to describe accurately.

Our study confirms that the morphological species concepts of “*T. palmata*” in Asia, Europe, and North America are, in many cases, problematic, and the host preference and geographical distribution have not been given enough attention. The real host and distribution range of *T. palmata* is, in fact, more limited than the descriptions in the literature. Similar cases have also been widely reported in other fungal groups, for instance, the *Amanita sculpta* complex (Huang et al., 2023), *Clavariadelphus amplus* complex (Huang et al., 2020), *Hygrophorus esculentus* complex (Huang et al., 2022), *Hygrophorus russula* complex (Huang et al., 2021), and *Rhodotus palmatus* complex (Tang et al., 2014). Therefore, careful observation of habitat as well as recording odor and potential partners is essential in the taxonomy of ectomycorrhizal fungi. Hopefully, the recognition that there is more than one species in the *T. palmata* complex will provide an incentive for future collectors to record detailed macromorphological and habitat information.

## Data Availability

The datasets presented in this study can be found in online repositories. The names of the repository/repositories and accession number(s) can be found below: https://www.ncbi.nlm.nih.gov/genbank/, PQ504921 https://www.ncbi.nlm.nih.gov/genbank/, PQ538458 https://www.ncbi.nlm.nih.gov/genbank/, PQ560959 https://www.ncbi.nlm.nih.gov/genbank/, PQ538484 https://www.ncbi.nlm.nih.gov/genbank/, PQ560960 https://www.ncbi.nlm.nih.gov/genbank/, PQ538459 https://www.ncbi.nlm.nih.gov/genbank/, PQ504922 https://www.ncbi.nlm.nih.gov/genbank/, PQ504919 https://www.ncbi.nlm.nih.gov/genbank/, PQ538456 https://www.ncbi.nlm.nih.gov/genbank/, PQ560957 https://www.ncbi.nlm.nih.gov/genbank/, PQ538482 https://www.ncbi.nlm.nih.gov/genbank/, PQ538483 https://www.ncbi.nlm.nih.gov/genbank/, PQ560958 https://www.ncbi.nlm.nih.gov/genbank/, PQ538457 https://www.ncbi.nlm.nih.gov/genbank/, PQ504920 https://www.ncbi.nlm.nih.gov/genbank/, PQ504940 https://www.ncbi.nlm.nih.gov/genbank/, PQ538477 https://www.ncbi.nlm.nih.gov/genbank/, PQ504935 https://www.ncbi.nlm.nih.gov/genbank/, PQ538472 https://www.ncbi.nlm.nih.gov/genbank/, PQ560963 https://www.ncbi.nlm.nih.gov/genbank/, PQ538497 https://www.ncbi.nlm.nih.gov/genbank/, PQ504936 https://www.ncbi.nlm.nih.gov/genbank/, PQ538473 https://www.ncbi.nlm.nih.gov/genbank/, PQ560974 https://www.ncbi.nlm.nih.gov/genbank/, PQ504931 https://www.ncbi.nlm.nih.gov/genbank/, PQ538468 https://www.ncbi.nlm.nih.gov/genbank/, PQ560969 https://www.ncbi.nlm.nih.gov/genbank/, PQ538493 https://www.ncbi.nlm.nih.gov/genbank/, PQ504932 https://www.ncbi.nlm.nih.gov/genbank/, PQ538469 https://www.ncbi.nlm.nih.gov/genbank/, PQ560970 https://www.ncbi.nlm.nih.gov/genbank/, PQ538494 https://www.ncbi.nlm.nih.gov/genbank/, PQ504938 https://www.ncbi.nlm.nih.gov/genbank/, PQ538475.
