# Supplementary figures and images for: Multi-loci phylogeny reveals unexpected novelty of the Thelephora palmata complex (Thelephoraceae, Thelephorales) from China
Source: Front Fungal Biol. 2025 Aug 14;6:1599905. doi: 10.3389/ffunb.2025.1599905 (PMC12391122; doi:10.3389/ffunb.2025.1599905)

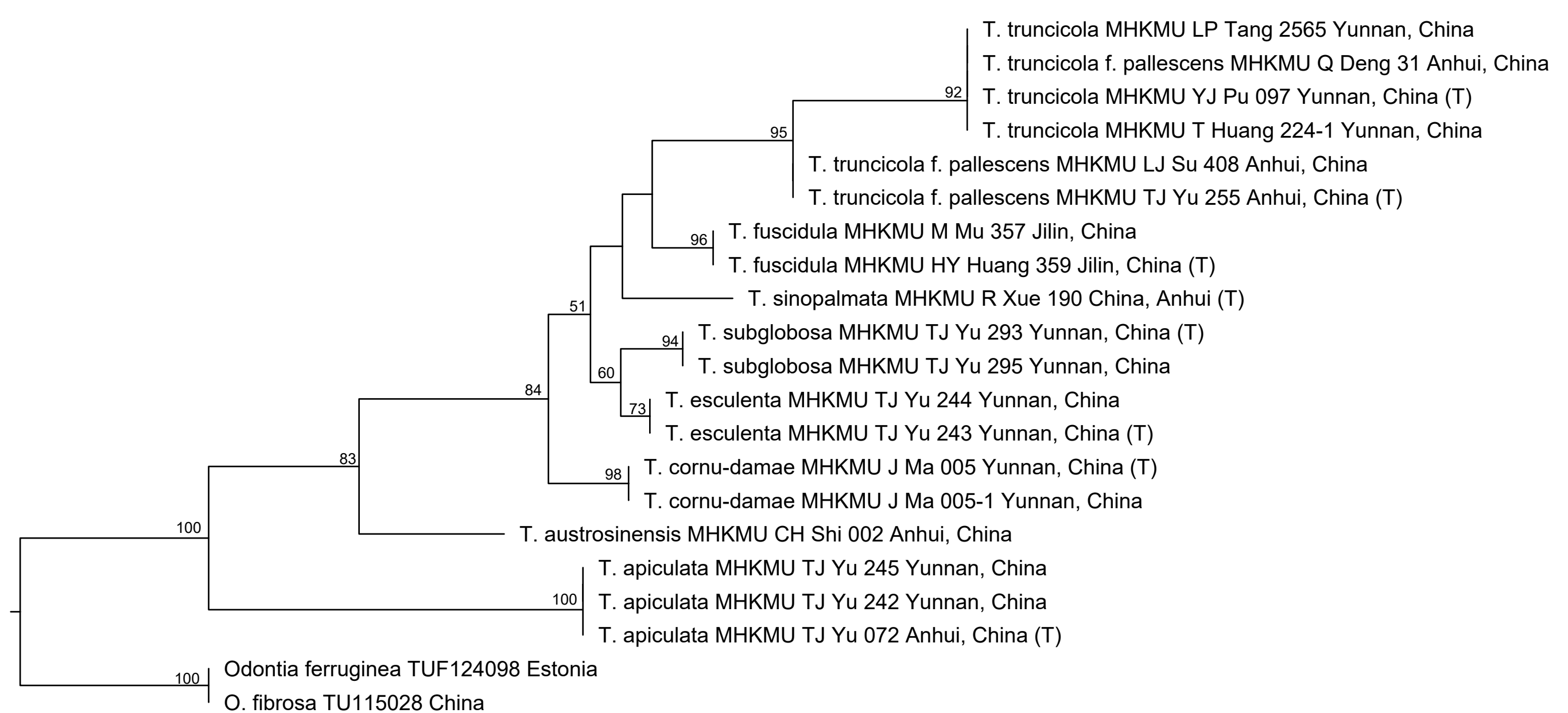

Supplement: Supplementary file 1 [file DataSheet1.pdf]

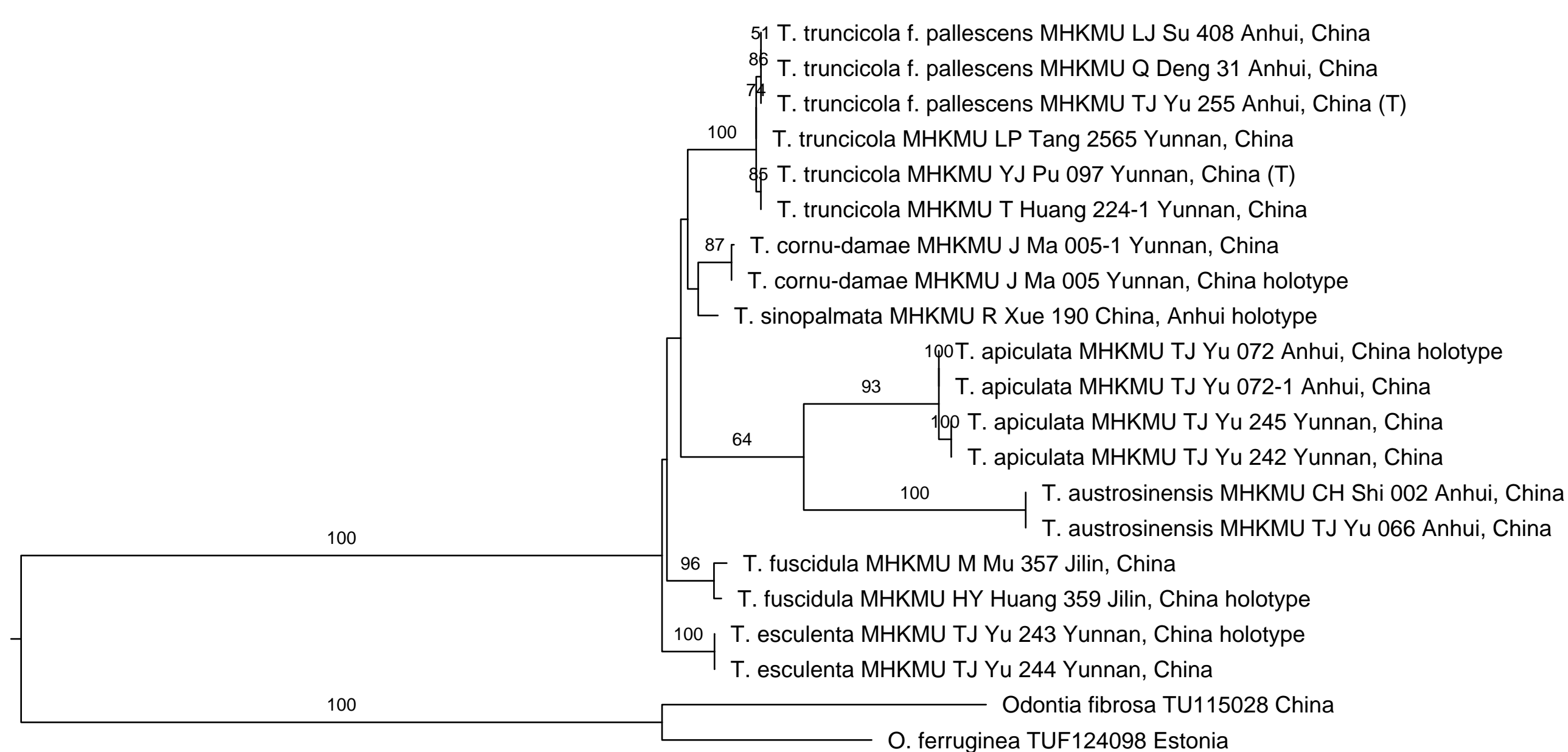

0.06

Supplement: Supplementary file 2 [file DataSheet2.pdf]
